# The word and the way: strategies for domain-specific BERT pre-training in German medical NLP

**DOI:** 10.1186/s12911-026-03728-2

**Published:** 2026-08-06

**Authors:** Henry He, Johann Frei, Raphael Schmitt

**Affiliations:** 1 School of Computation, Information and Technology, Technical University of Munich, Munich, Germany; 2 Chair of IT Infrastructure for Translational Medical Research, Faculty of Applied Computer Science, University of Augsburg, Augsburg, Germany; 3 Institute of General Practice, Faculty of Medicine and Medical Center, University of Freiburg, Freiburg, Germany

**Keywords:** Natural language processing, Medical informatics, Machine learning, Electronic health records, Named entity recognition, Text classification, Language models, Biomedical text mining, Germany

## Abstract

**Background:**

Digital healthcare generates vast amounts of clinical texts that hold potential for AI-assisted applications. However, existing German biomedical language models either rely on older architectures or are trained on limited data, which may hinder their performance in real-world settings.

**Methods:**

To explore the impact of domain adaptation strategies in German clinical NLP, we developed a family of domain-specific RoBERTa-based language models, collectively referred to as *ChristBERT* (**C**linical- and **H**ealthcare-**R**elated **I**ssues and **S**ubjects **T**uned BERT). To address the lack of large-scale German clinical corpora, we curated a 13.5 GB dataset consisting of scientific publications, clinical texts, and health-related web content. Additionally, we employed data augmentation via translation of English clinical corpora. Three domain adaptation strategies were explored: continued pre-training, pre-training from scratch, and pre-training with domain-specific vocabulary adaptation.

**Results:**

The resulting models were evaluated on three medical named entity recognition and two text classification tasks. Our models consistently outperformed four existing general-purpose and medical German models on four out of five tasks. The results demonstrate that the choice of domain adaptation strategy significantly influences downstream task performance. Based on the empirical results, pre-training from scratch is effective for highly specialized clinical texts, whereas continued pre-training is suited for more commonly written medical texts.

**Conclusions:**

ChristBERT establishes a new state-of-the-art for German clinical language modeling. Our findings indicate that the optimal domain adaptation strategy is task-dependent and remains crucial, as adapted models consistently outperformed general-purpose language models in our experiments. To support further research and application in German medical NLP, all developed models are publicly released.

**Supplementary Information:**

The online version contains supplementary material available at 10.1186/s12911-026-03728-2.

## Introduction

The digitization of health services and clinical processes has resulted in the healthcare industry generating an ever-increasing amount of textual data, encompassing electronic health records, clinical notes, medical reports, and discharge letters among many others. While structured data is frequently used for health economics and registries, the aforementioned unstructured clinical narratives are preferred by physicians to record patients’ clinical information due to their flexibility and efficiency, and account for up to 40% of the data generated in current hospital systems [1, 2]. The substantial potential of narrative text data to support clinical applications was recognized early [3–5] and more recently, research efforts have been directed towards developing medical applications assisted by artificial intelligence (AI). Prominent applications include decision support systems that assist healthcare professionals in their tasks, alleviating their workload and providing better treatments for patients [6].

However, the unstructured nature of textual data and the intricacies of the biomedical field pose significant challenges for leveraging its potential. In such a context, Natural Language Processing (NLP) methods could structure that information to support downstream clinical applications. Recent advancements in NLP brought about by large-scale pre-trained language models based on the Transformer [7] architecture have introduced new ways for extracting and analyzing the knowledge contained within the clinical texts. Through extensive self-supervised training on vast corpora of text, a model can acquire valuable representations of a language, producing highly effective language models.

The success of Transformer-based models like Bidirectional Encoder Representations from Transformers (BERT) [8] and its improved version Robustly optimized BERT Pretraining approach (RoBERTa) [9], can be largely attributed to the use of transfer learning expressed in the pretrain-finetune paradigm. In this paradigm, a model initially goes through a resource-intensive training process, i.e. *pre-training*, using general-purpose textual data to learn the language structure. This pre-training phase is self-supervised, eliminating the need for labeled data by utilizing objectives like masked language modeling [8]. The model is then *fine-tuned* for various tasks through a second, more cost-effective training round using a smaller, labeled, and task-specific dataset that adjusts the model’s weights to fit the specific task and application domain at hand.

Direct application of general-purpose language models to a specific domain might limit performance due to significant distributional differences between general and target domains. Even within the same language, domain-specific language can vary significantly from everyday language, leading to the need for domain-specific models [10]. This particularly holds for the medical domain, where the language is highly specialized and complex. Medical language features numerous acronyms that are crucial for saving time and space, yet they can be ambiguous and require context to be understood. Spelling errors are common, and there is an abundance of abbreviations [11]. Moreover, the medical vocabulary is highly specialized, as it is not typically used in everyday language, making it unfamiliar to those outside the medical profession. When the target domain, such as medicine, differs considerably from the pre-training data, models can be improved by an additional phase of domain-adaptive training using large, domain-specific corpora with the same pre-training objectives. Similar strategies have previously been explored in English biomedical NLP through models such as BioBERT [12], SciBERT [13], and PubMedBERT [14], which differ in their use of continued pre-training and training from scratch.

Such specifically designed medical language models hold significant promise for enhancing the efficiency and precision of medical document handling [12, 13, 15, 16]. For the German medical domain, the effectiveness of such models has been demonstrated by BioGottBERT [17] and medBERT.de [18]. However, the availability of open-source biomedical corpora large enough for domain adaptation is limited, primarily due to the sensitive nature of health-related data, and is largely confined to the English language, given its established status as the language of science. Despite these obstacles, advancing medical language models remains crucial, as they have the potential to manage the large volumes of text produced in hospitals every day. In this work, we aimed to develop a new comprehensive German clinical language model based on the RoBERTa architecture by building upon the foundation laid by GeistBERT [19], hereinafter referred to as *ChristBERT*: **C**linical-and **H**ealthcare-**R**elated **I**ssues and **S**ubjects **T**uned BERT. The main emphasis of this work lies in the construction of a large German pre-training corpus, encompassing a diverse range of biomedical and clinical texts. These sources provided a broad spectrum of medical language data, fostering the model’s robustness and applicability. In order to achieve this, we utilized a combination of mostly publicly available German medical textual data and synthetic German domain texts by augmenting the corpus with translated medical texts [20]. This approach involves translating a monolingual corpus using neural machine translation models [21, 22], allowing us to leverage the vast amount of public English medical texts available. Based on the constructed corpus, we pre-trained ChristBERT by using Whole Word Masking (WWM) and following three different domain-adaptation strategies: (1) continued pre-training, (2) pre-training from scratch with general-purpose vocabulary, and (3) pre-training from scratch with additional prior vocabulary adaptation. In order to investigate the effects of the different domain-adaptation approaches, we evaluated the performance of the resulting models on two domain-specific downstream tasks: named entity recognition and classification. The downstream task performance has been thoroughly evaluated and compared to existing medical and general-purpose German language models.

## Related work

Past developments in medical NLP research have seen the creation of mature systems for extracting information from English clinical texts like MetaMap [23], cTAKES [24], MedLEE [5, 25] and CLAMP [26]. These systems have been used for various tasks such as Named Entity Recognition (NER), relation extraction, and information retrieval. Additionally, open competitions such as Informatics for Informatics for Integrating Biology and the Bedside (i2b2) [27], National NLP Clinical Challenges (n2c2) [28, 29], and CLEF eHealth [30] challenge from the Conference and Labs of the Evaluation Forum (CLEF) promote data and model sharing, further advancing the medical NLP field. The systems developed to date encompass rule-based, machine-learning-based, and hybrid models. While rule-based methods were essential in early developments, the performance of these systems is limited by their reliance on hand-crafted rules and lexicons, which are difficult to maintain and generalize across different clinical settings.

In order to overcome these challenges, current research emphasizes machine-learning techniques. In particular, deep-learning approaches like recurrent neural networks (RNNs) and convolutional neural networks have been widely used in recent years due to their ability to achieve superior performance with adequate training data. Unlike traditional machine-learning methods, deep neural networks typically use methods such as Word2Vec [31], GloVe [32], or FastText [33] to represent words as vectors. These methods create word embeddings by learning relationships between words from large text corpora, eliminating the need for manual feature engineering. Nevertheless, these methods represent all possible meanings of a word in a single vector, making them unable to distinguish between different word senses based on the surrounding context. Vaswani et al. [7] introduced a new model, the Transformer, capable of producing contextualized word representations. Originally designed for neural machine translation, the Transformer addresses two limitations of RNNs: lack of parallelization and handling of long-range dependencies. It relies on the self-attention mechanism, which differentially weighs parts of the input. Since it operates without recurrence, it is more parallelizable and computationally efficient than RNNs.

In 2019, Devlin et al. [8] utilized parts of the original Transformer architecture to develop BERT, achieving state-of-the-art results in numerous NLP tasks. Performance of these large-scale language models heavily depends on the underlying data used for pre-training. A homogeneous text corpus generally leads to a poorer performing model compared to one trained on diverse text corpora of high variance [34]. Initially, much of BERT research was conducted with English texts, followed by efforts in multilingual approaches [35]. While multilingual models were trained on extensive texts from numerous languages, it has been shown that single language models outperform these and offer advantages in efficiency, pre-training efforts, and downstream task performance as they demand fewer computational resources and smaller datasets compared to the extensive and diverse data required for multilingual models [34, 36, 37]. In particular, single-language models trained with the Open Super-large Crawled ALMAnaCH coRpus (OSCAR) [38] demonstrated strong performance, benefiting from the corpus’s size and variability. Notable examples include CamemBERT [34] for French, GottBERT [36] for German, and BERTje [39] for Dutch.

With the increasing use of Transformer-based models in NLP, there is a growing need in the clinical domain for language models that are not only accurate but also efficient, resource-conscious, and suitable for local processing. In settings with limited computational resources and strict data privacy requirements, small yet high-performing domain-specific models can provide substantial benefits. Continued pre-training on in-domain data has proven effective for enhancing performance on specialized clinical tasks. In the biomedical field, the pioneering and most recognized pre-trained model is BioBERT [12], which shares the same architecture as BERT. Following a domain-adaptation strategy, BioBERT starts with BERT weights pre-trained on general texts and then refines these weights using biomedical corpora, surpassing the original model and achieving state-of-the-art performance in numerous biomedical text mining tasks, such as clinical concept recognition, gene-protein relation extraction, and biomedical question answering. To gather sufficient open-source biomedical data, the authors utilized repositories like PubMed [40] and PMC [41], obtaining 4.5 billion words from abstracts and 13.5 billion words from full-text articles. A similar method is employed by SciBERT [13], which retains the original BERT configuration but substitutes the initial general corpora with 1.14 million scientific articles randomly chosen from Semantic Scholar. This dataset consists of 82% broad biomedical domain papers and 18% computer science domain papers. By training from the ground up on biomedical data, SciBERT can utilize a custom dictionary that better represents the domain-specific word distribution. Med-BERT [42] is the first model fully trained on hospital data, particularly semi-structured electronic health records, leading to enhanced performance in subsequent prediction models. These approaches have since been refined, either by updating the model architecture to use BERT variants or by expanding the biomedical corpus with additional sources beyond scientific literature [15, 16].

The extensive range of biomedical and clinical BERT-based models benefit from the abundance of publicly available biomedical data in English, such as MIMIC [43, 44], the largest open-access dataset of medical records, and extensive repositories of biomedical scientific literature [40]. However, most other languages lack access to these valuable resources, making it challenging to achieve the same level of performance as their English counterparts. Despite this, researchers from various countries have endeavored to pre-train non-English biomedical models, utilizing local and often non-public biomedical text collections. They have either trained new models from scratch [45] or applied biomedical domain adaptation to multilingual [46] or monolingual [47] versions of BERT.

For what concerns the German language, advancements in medical language models are significantly delayed and are often propelled solely by commercial software or localized applications [48]. Stringent data protection laws impede data sharing, leading clinics to restrict data usage to internal purposes [49]. These obstacles hinder the sharing of datasets and models, as well as the organization of open challenges involving German datasets. In spite of these challenges, there have been notable initiatives in recent years: Datasets such as Jena Synthetic Clinical Case Corpus (JSynCC) [50] and German Guideline Program in Oncology NLP Corpus (GGPONC) [51] have been released, containing German biomedical language texts that are not subject to data protection concerns. Recently, the introduction of the Berlin-Tübingen Oncology Corpus (BRONCO150) [52] corpus, which includes de-identified discharge letters, and GPTNERMED [53], which leverages large language models, has further expanded the availability of German medical text data. Additionally, the CLEF eHealth challenge in 2019 provided a dataset of non-technical summaries of animal studies to be classified according to the International Classification of Diseases and Related Health Problems (ICD-10) [54–56]. A study by [57] utilized the multilingual BERT (mBERT) version to classify these summaries, demonstrating that mBERT significantly outperformed a baseline Support Vector Machine model. To incorporate advances in general German language models [17], introduced BioGottBERT, a model pre-trained on open medical German texts from Wikipedia and scientific abstracts, which demonstrated superior performance over its generalized counterpart GottBERT on medical tasks. Subsequently, the authors of [18] proposed medBERT.de, in order to address the limited training data size and narrow scope on merely one medical subarea by using 3.8 million radiology reports, achieving promising results in classification tasks. While BioGottBERT was trained on a relatively small corpus of slightly less than 1 GB of text, medBERT.de significantly expanded its training corpus to 10 GB, incorporating a wider variety of sources. However, its BERT architecture has since been superseded by its optimized variant RoBERTa, as recently demonstrated for German by the GeistBERT model [19]. GeistBERT built upon GottBERT [36], by using WWM and continued pre-training on a significantly more varied and larger general-domain corpus, thereby establishing state-of-the-art performance on various German NLP benchmarks.

## Methodology

### Corpus creation

Main shortcomings of existing German medical domain models include the limited availability of training data due to the sensitive nature of medical information and strict data privacy regulations. Furthermore, many existing biomedical Transformer models [17, 18] are pre-trained or evaluated on proprietary datasets, hindering independent model verification and validation. Previous studies [18, 34, 58] concluded that training data diversity and quantity are more important than excessive data cleaning, which had a negligible effect on downstream performance. Following these findings, we compiled a 13.5 GB large and highly varied German biomedical and clinical corpus, focusing on data quantity over quality. In order to mitigate the aforementioned shortcomings, we primarily relied on public datasets with only two private data sources included, to foster transparency and accessibility of the ChristBERT models. Table 1 summarizes the pre-training corpus, including descriptive statistics about the number of documents, sentences, words, and size of each incorporated dataset.

**Table 1:** Overview of datasets contained in the pre-training corpus. The table provides details about each dataset, including the number of documents, sentences, words, and their size in megabytes. The final corpus includes all listed datasets and amounts to roughly 13.5 GB of pre-training data

Dataset	# Documents	# Sentences	# Words	Size (MB)
**Native German corpora**				
Hpsmedia	277,357	16,314,452	405,316,578	3,117
Springer Nature	258,000	14,158,151	259,284,884	1,984
PhD Theses	7,486	4,665,850	90,380,880	646
Medical Wikipedia	75,585	3,254,135	49,594,111	362
Web Crawl	93,642	4,024,816	68,797,358	512
**Translated corpora**				
PubMed Central	90,273	8,644,017	220,033,966	1,609
MIMIC-IV Notes	330,486	49,351,295	733,952,748	5,310
Summary	1,132,829	100,412,716	1,827,360,525	13,540

#### Hpsmedia

Hpsmedia is a German publisher specializing in medical content primarily targeted at healthcare professionals. Hpsmedia publishes three healthcare journals: *Pflegewissenschaften (Nursing Sciences)*, *Pädagogik der Gesundheitsberufe (Pedagogy of Health Professions)* and *Geschichte der Gesundheitsberufe (History of Health Professions)*, which are available in print and online. All journals publish articles in German and are peer-reviewed by experts in the respective fields, following the BMJ peer-review guidelines [59]. The articles cover a wide range of topics within the healthcare domain including aspects of health and nursing care, pedagogy, didactics, curricula, education in healthcare professions and the history of healthcare professions. We were kindly provided with the full-text content of the journals in CSV format by Hpsmedia. The CSV files were processed using the Pandas [60] Python library to extract the text content of the articles, which was then included in the pre-training corpus. The Hpsmedia dataset consists of 277,357 documents totaling to 3,117 MB of data.

#### Springer nature

Springer Nature is a prominent global publisher of academic content, known for its extensive collection of high-quality journals, books, and research materials across various disciplines, including science, technology, and medicine.

For the extraction of text from Springer Nature publications, the Springer Nature API [61] was utilized. The API offers multiple endpoints, e.g. metadata, full-text (TDM) as well as a wide range of constraint parameters to filter for desired publications, which are returned in XML format. This allowed for a systematic filtering for open-access publications in German. For our purposes, the open-access API was first queried for metadata of articles and books related to the subjects of *biomedicine*, *public health*, *pharmacy*, *dentistry* and *life sciences*. The returned XML data was then processed to extract abstracts and Digital Object Identifiers (DOIs) of each publication, respectively. Subsequently, the set of DOIs was used to make bulk API calls to the TDM endpoint to fetch the full-text content of the publications. In a final step, the extracted abstracts and full-text content were both incorporated into the pre-training corpus accounting for a total of 258,000 documents and 1,984 MB of data.

#### PubMed central

**Fig. 1:**
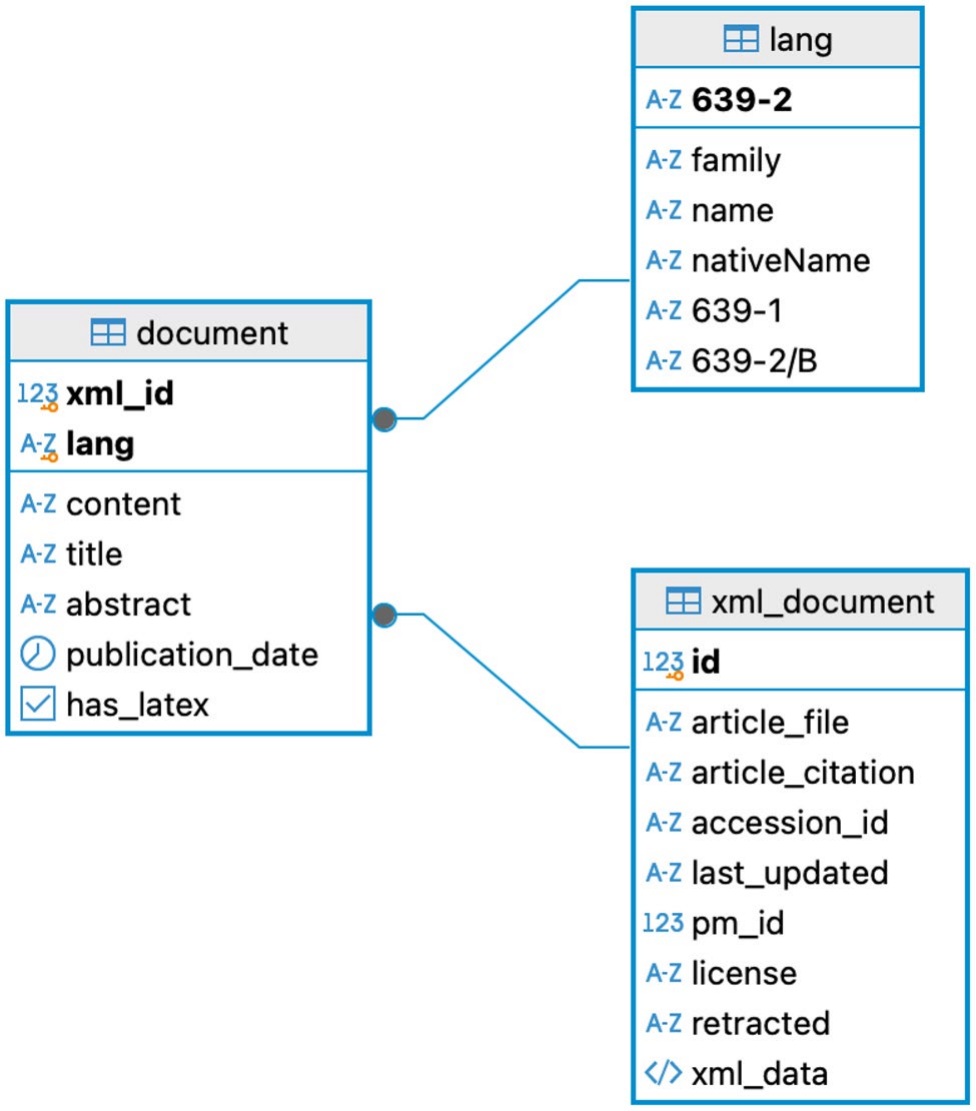
The diagram shows the database relations for translation management. The raw XML files with their metadata are stored in the **xml_document** table, while the **document** table contains the extracted text data with both PubMed ID and the language of a document as its primary keys. Languages are foreign keys to the corresponding entity in the **lang** relation according to the ISO 639–3 standard

PubMed Central (PMC) is a free digital repository of full-text scientific literature in the field of biomedicine and life sciences, created as an extension of PubMed [40], which holds bibliographic references and abstracts for essentially all publications in the biomedical sciences. Both repositories are maintained by the National Center for Biotechnology Information (NCBI), a part of the United States National Library of Medicine (NLM). The PMC archive provides access to a collection of over 10 million research articles, reviews, and other scientific publications from a wide range of biomedical and life science journals. Not all articles in PMC are available for text mining or other reuse, as many are under copyright. The *PMC Open Access Subset* [41] contains those articles made truly freely available to the public under Creative Commons or similar licenses that allow more liberal redistribution and repurposing than the majority of licensed and copyrighted articles from subscription access journals deposited in PMC. PMC stores content in XML format, which is structured according to the Journal Article Tag Suite (JATS) standard, a widely used archival markup format for journal articles. The JATS XML files are made available by NLM for bulk download through their PMC FTP Service [41]. We downloaded the December 2024 baseline package of the Commercial Use Collection of the PMC Open Access Subset and transferred the XML files with appropriate metadata such as PubMed ID and publication date to a PostgreSQL database for further processing. The database design is shown in Fig. 1, which is represented by an entity-relationship diagram. The XML files and their corresponding metadata are stored in the **xml_document** table by leveraging the native support of PostgreSQL for XML data types. For our needs, we extracted the title, abstract, full-text content and language of the articles from the XML markup by utilizing the Pubmed Parser [62] Python library, which supports parsing of the JATS XML format. The extracted text data was then stored in the **document** table, which contains the PubMed ID and language of each document as its primary keys. The language of each document is represented as a foreign key to the **lang** table, which contains the ISO 639–3 language codes. The **lang** table is used to ensure data integrity and consistency across the database.

**Fig. 2:**
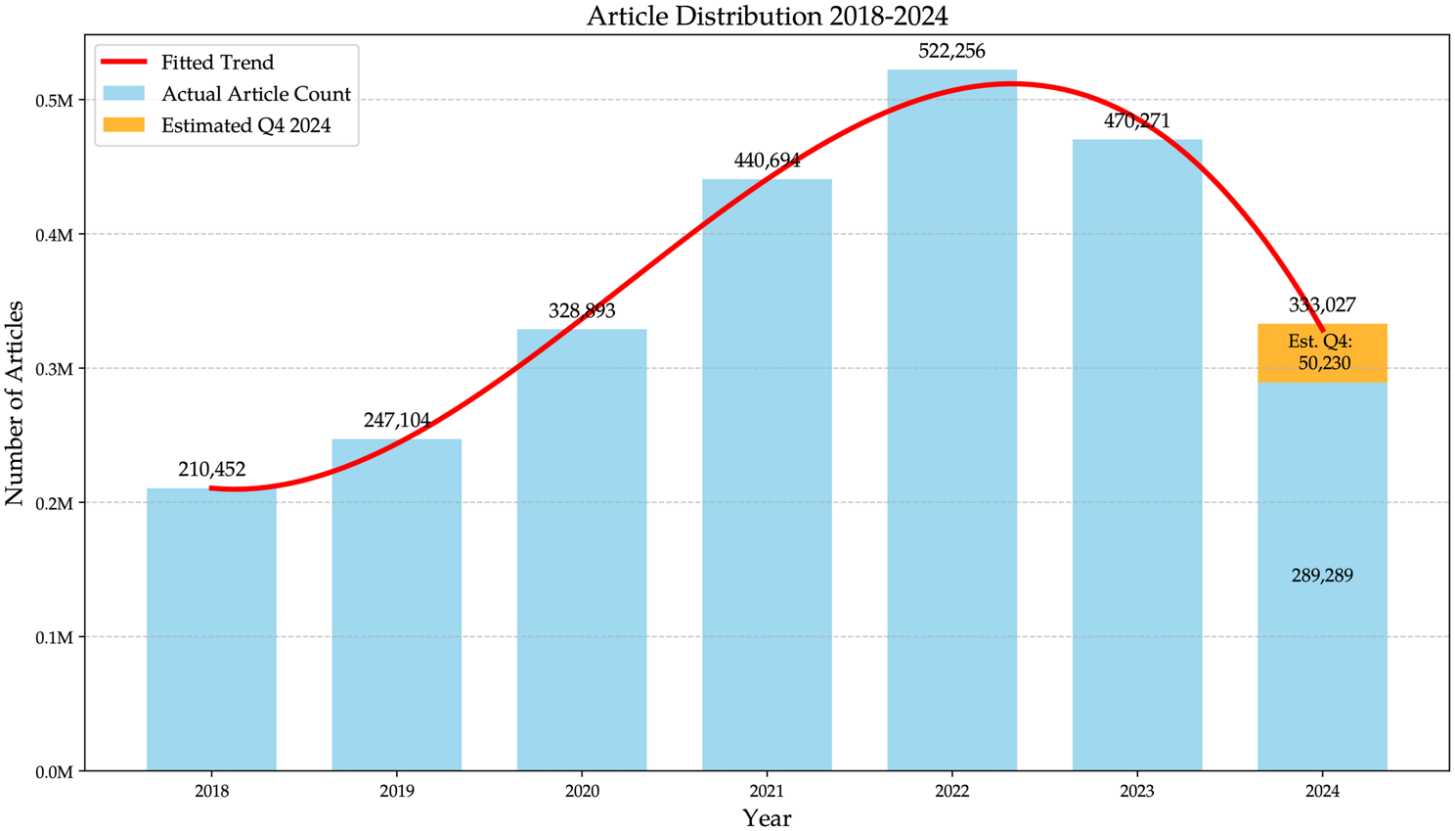
This histogram shows the annual number of articles published in PMC from 2018 to 2024, with a fitted trend line indicating the overall growth and decline of article counts. The estimated article count for Q4 2024 was approximated based on the maximum Q4 article count observed in previous years (50,230)

**Fig. 3:**
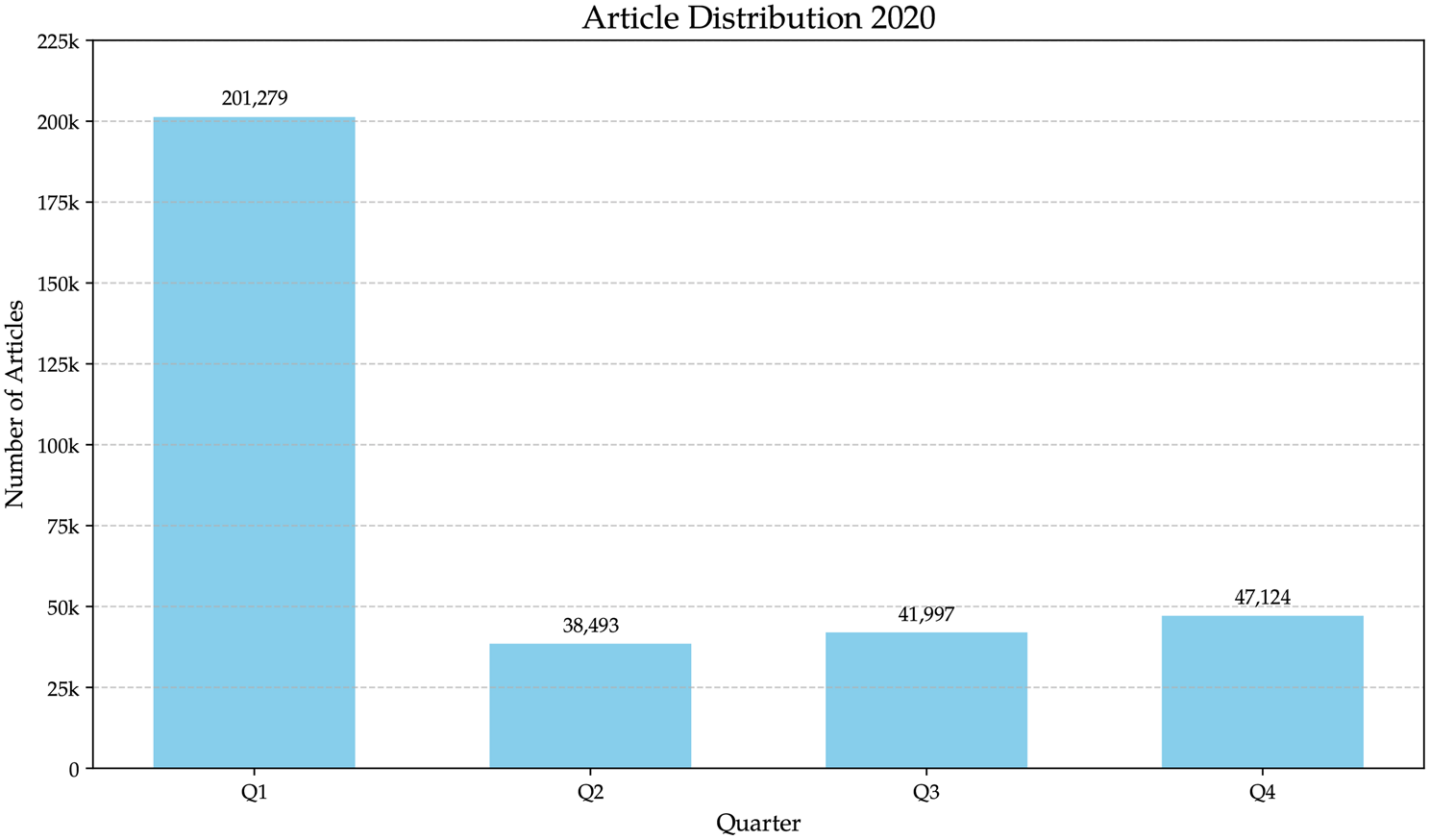
The histogram shows the quarterly number of articles published in PMC in 2020, with a peak in Q1 at 201,279 articles, followed by a drop in the subsequent quarters

**Fig. 4:**
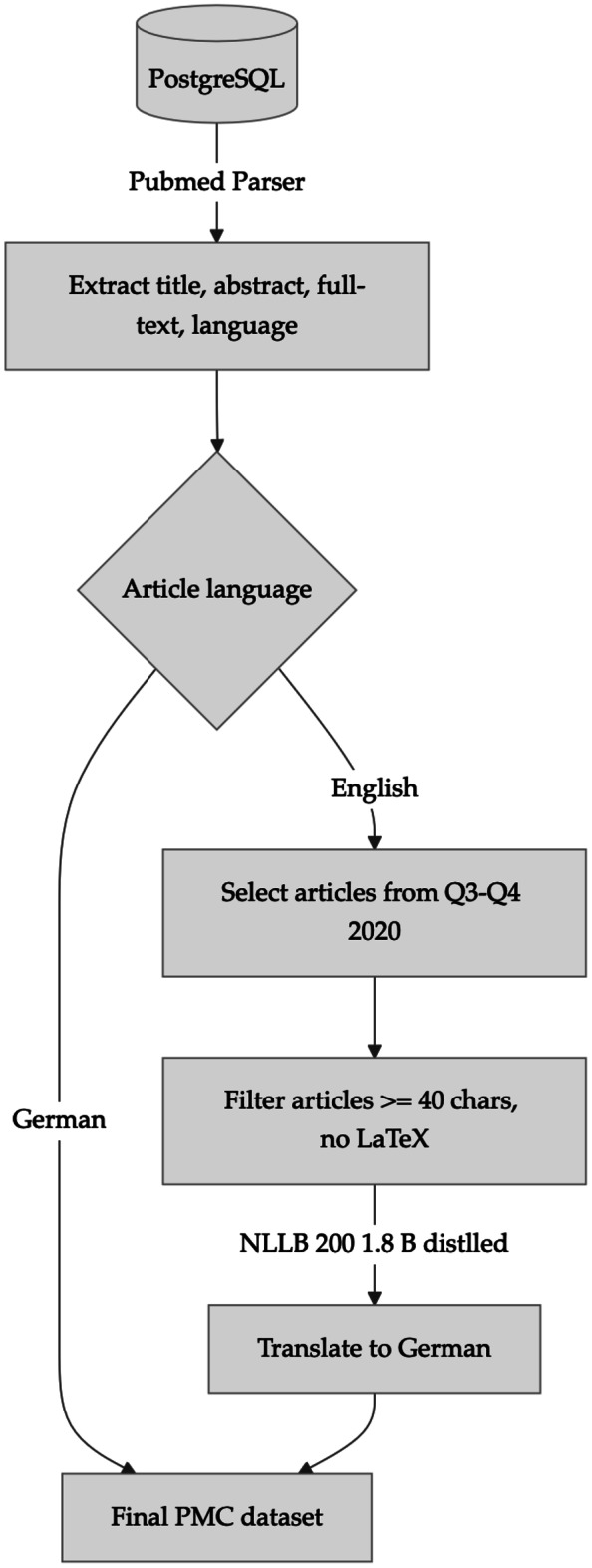
This flowchart illustrates the sequential steps involved in the translation process of articles from PMC. It begins with extracting article details from PostgreSQL using Pubmed Parser, followed by language-based filtering to select English articles from Q3 and Q4 of 2020. Articles are then filtered based on length and $\hbox{\LaTeX}$ content, followed by translation to German using the NLLB 200 1.3B distilled model

In order to leverage the large amount of English-language content available in PMC, we translated the English articles to German using the NLLB 200 [22] neural machine translation model in its 1.3 billion distilled variant. Translation was performed on two Nvidia GeForce RTX 3090 24 GB GPUs, while leveraging the *NLLB-API* [63] library for parallel processing. The translation posed a significant computational challenge, which was addressed by limiting the publications to be translated to those published in the third and fourth quarters of 2020. This decision was based on an analysis of article distribution over the past seven years, which is depicted in Fig. 2. The analysis revealed a notable peak in publications in 2022, potentially influenced by the COVID-19 pandemic and the emergence of generative AI. To ensure that the translated content was not overly biased towards the COVID-19 pandemic and mitigate the presumably uniform writing style resulting from generative AI, we selected the year 2020 for our translations. Likewise, given our computational constraints, we chose the third and fourth quarters of 2020, as the quarterly distribution of articles in that year indicated a more feasible volume of publications as seen in Fig. 3. Translated documents were saved back to the database in the **document** table, but with an updated language key set to **de** for German. Further data filtering encompassed the removal of articles with fewer than 40 characters and those containing $\hbox{\LaTeX}$ markup. Fig. 4 summarizes the described steps for PMC translation as a flowchart. The translated and natively German articles were then combined into a single dataset, resulting in a total of 90,272 documents and 1,609 MB of text data.

#### PhD theses

In this work, we also included a collection of 7,486 open-access German-language dissertations and postdoctoral dissertations from Charité University Hospital, Germany’s largest university hospital. At the joint medical faculty of Humboldt University and Free University of Berlin, electronically published documents, doctoral and habilitation theses, as well as research data are made available to the public through the university’s institutional repository Refubium [64]. The documents were downloaded in bulk as PDF files and subsequently converted to plain text. Data cleaning involved removing sentences that lacked German stop words and excluding theses under 15 pages in length. This process ensured the inclusion of only relevant, high-quality text data. In total, 646 MB of text was extracted from the PhD theses.

#### Medical wikipedia

Wikipedia curates entry pages on the encyclopedia about particular subject areas in so-called *portals*. Each portal acts as a hub, bringing together key articles, images, and resources about the respective topic. Portals are particularly useful for getting an organized overview or exploring related subtopics without searching through individual articles. We utilized the German Wikipedia portal on medicine in order to extend our pre-training corpus with freely available texts on medical topics, which were collectively authored and editorially proofread by a diverse community of volunteer contributors. Wikipedia does not offer an API for bulk data retrieval, but instead provides an export interface [65] for downloading specified wiki pages in a special XML format. These XML files follow a schema specific to MediaWiki, the software behind Wikipedia, initially intended for importing into another MediaWiki installation but also allows for further processing and analysis.

The export interface expects either a list of page titles or a category name, which it resolves into a list of pages related to the given category. Since our objective is to crawl the entirety of the medical portal, we implemented a breadth-first search algorithm on Wikipedia’s export interface, employing Selenium WebDriver [66] to traverse the category tree of the portal. The algorithm starts at the root category **Portal:Medizin** and recursively visits each subcategory, collecting the titles of all pages contained within. The page titles are then used to download the corresponding XML files in bulk, creating a dump of the entire German medical portal. The MediaWiki XML files are parsed using Python’s **ElementTree** module to extract page contents. Wikitext formatting is then removed using **MediaWiki Parser from Hell** [67], resulting in clean plain text documents. The German Wikipedia portal on medicine contributes a total of 75,585 documents and 362 MB of data to the pre-training corpus.

#### MIMIC-IV notes

Medical Information Mart for Intensive Care IV (MIMIC-IV) [43] is a large and freely accessible electronic health record dataset comprising various health-related data acquired during routine clinical care of patients admitted to critical care units of the Beth Israel Deaconess Medical Center in Boston, MA, USA. MIMIC-IV constitutes the fourth edition of the dataset, containing data of over a decade from 2008 to 2019 and covering a wide range of information such as patient measurements, orders, diagnoses, procedures, treatments, and clinical notes.

For our corpus, we specifically chose to use the *clinical notes* [44] subset of the MIMIC-IV database as it is made up of discharge summaries written in the form of free text, which is well suited for training contextual language models. The 330,485 discharge summaries from 145,915 hospitalized patients are organized into sections including chief complaint, history of present illness, past medical history, brief hospital course, physical exams, and discharge diagnoses. These free-text notes were acquired from the hospital system and de-identified by the authors using a combined automatic approach of custom rules and a neural network trained on de-identification, cast as a NER task.

The note subset of the MIMIC-IV dataset is available on *PhysioNet* [68], a repository for freely accessible medical data and tools for computational medicine research. After downloading the collection of clinical notes, we utilized LLMs to translate the English discharge summaries to German. Specifically, we employed the multilingual *LLaMA 3.1 8B* [69] model in an API-like manner by providing the prompt as shown in Table 2. The translated notes were then included in the pre-training corpus, consisting of 330,485 documents and 5,310 MB of data. The choice of translation model was guided by the characteristics of the respective corpora. While NLLB-200 provided an efficient solution for large-scale translation of structured scientific prose in PMC, the instruction-following capabilities of LLaMA 3.1 were particularly suitable for translating heterogeneous clinical narratives while preserving pseudonymization masks through explicit prompting.

**Table 2:** LLaMA 3.1 system prompt and user instruction used for MIMIC-IV translation

System Prompt:
You are an API-like assistant, and output only the plain response without further explain or comment the output.
**User Instruction:**
Translate the following text strictly into German. Do not replace the___ pseudonymization masks. * < *English Text* > *

#### Web crawl

To enrich our corpus with current medical content from the German web, a web crawl was performed using the implementation described in [70], which extends the open-source crawler Apache Nutch [71]. The crawl was seeded with a combination of domains from the *tala-med search* [72] index as well as the seed sources provided by the *sampled German Health Web* (sGHW) [73] project. Tala-med search is a specialized search engine that provides high-quality, evidence-based health information. In its current version, it indexes 26 trustworthy German health websites and ensures strict user privacy. The sGHW project represents previous efforts to index health-related web content in the German language and employed a specialized focused crawler to create an index of 22,405 German health websites. The sGHW index was limited to websites with. **de**, .**at**, and .**ch** top-level domains, and used a support vector machine to filter content for health relevance automatically. Our crawl was configured with parameters **depth = 3** and topN = 100. In web crawling, **depth** refers to the number of hops or iterations the crawler will follow links from the seed URL, while the width, called **topN**, specifies the maximum number of URLs to fetch in each iteration. These parameters control the crawling process and were chosen to allow for systematic exploration of linked content while maintaining a manageable scope.

Despite the focused seed list, unsuitable as well as nonmedical content, such as advertisements, remained present in the crawl data due to the nature of web crawling. To address this issue, we developed a text classifier in order to filter medical and scientific content from general web content, ensuring the relevance of the gathered data. The classifier was built by fine-tuning GeistBERT on a binary-labeled dataset derived from the scientific portion of the *10kGNAD* [74] corpus. The 10kGNAD dataset is a subset of the *One Million Posts Corpus* [75] and consists of 10,000 German news articles, including 573 focused on scientific topics. These scientific articles make up the first half of the fine-tuning dataset, while the second half was created using a stratified sample to ensure a balanced dataset and that each category was proportionally represented. The classifier’s performance was evaluated using a manually labeled subset of the web crawl data of size 119, which was annotated using Label Studio [76], an open-source data labeling tool. On this test set, the classifier achieved an F_1_ score of 80.34%, indicating a reliable level of accuracy. Following this validation, we applied the classifier to filter the complete web crawl dataset. After filtering, we removed documents from the web crawl with less than 40 characters, those containing the Unicode replacement character **U+FFFD** due to encoding issues, and duplicates. For the remaining documents, we removed phone numbers, email addresses, URLs, and emojis utilizing the clean-text [77] Python library. The preprocessing of the web crawl resulted in a final collection of 93,642 documents and 512 MB of data. Both the classifier [78] and the scientific subset used for training, referred to as *sciGNAD* [79], are publicly released to support reproducibility and downstream research. The collected web texts were used exclusively for research purposes as part of the pre-training corpus, whereas the original documents themselves are not redistributed as part of ChristBERT.

### Pre-training

Leveraging the created large-scale biomedical corpus as described in Section "Corpus creation" as well as the architectural foundation laid by the current state-of-the-art German general-purpose language model GeistBERT, we developed biomedical adaptations by following three main strategies:

1. **Continued pre-training**: Starting from the checkpoint of GeistBERT, we initialize a RoBERTa base model with the identical weights and general domain vocabulary. Subsequently, all parameters of the model are retrained on our 13.5 GB training data. Essentially, this approach is equivalent to extending GeistBERT’s pre-training dataset with the new data, which is why this strategy is known as *continued* pre-training. The created model following this approach will be referred to as ChristBERT.
2. **Pre-training from scratch**: We also explored the possibility of pre-training a RoBERTa model from scratch using the same architecture and vocabulary as GeistBERT, but without any initialization from the general domain model. As a result, this model solely learns language representations from our biomedical corpus. We denote this model as ChristBERT_scratch_.
3. **Vocabulary adaptation**: In order to study the impact of a domain-specific vocabulary, this strategy involves the creation of a new vocabulary based on the created biomedical corpus and follows the same pre-training process as ChristBERT_scratch_. The vocabulary is generated analogously to GeistBERT, using a GPT-2 style Byte- Pair Encoding (BPE) tokenizer with a target vocabulary size of 52,000 tokens. The resulting model is referred to as ChristBERT_BPE_.

Each of the three models was pre-trained using our fork of the fairseq [80] framework on the domain-specific corpus presented in Section "Corpus creation", which amounts to 13.5 GB of uncompressed text data. Data preprocessing was performed using our modular Snakemake-based framework and its accompanying utilities for GPT-2 BPE tokenization, generation of fairseq-compatible binary datasets, and model conversion [81]. The documents comprising the training data were shuffled in order to improve pre-training robustness. The models underwent training for 100,000 update steps with a batch size of 8,192, utilizing weight initialization based on one of the three previously outlined strategies. We adapted GeistBERT’s pre-training configuration, which closely aligns with RoBERTa’s standard training setup [9], encompassing dynamic masking for the WWM learning objective, *AdamW* optimizer parameters, and a fixed sequence length of 512 tokens. To comply with the maximum input sequence length of the model, full sentences from multiple documents in the pre-training corpus were packed into text segments. This procedure allows for retention of natural sentence structure despite the use of fixed-length sequences. For efficient data access, the fairseq library converts the input data into a binary format and utilizes memory-mapped file I/O. A warmup phase of 10,000 iterations was implemented, gradually increasing the learning rate to a maximum of $7\times10^{4}$ for ChristBERT and $6\times10^{-4}$ for ChristBERT_scratch_ and ChristBERT_BPE_, followed by a polynomial decay to zero. The complete pre-training procedure was performed on clusters equipped with either four Nvidia A100 interconnected via SXM or two Nvidia H100 GPUs. The cumulative training time for the three models amounted to approximately 21.7 days (refer to Table S2 in the Supplementary Material).

### Language modeling evaluation

To assess the impact of different pre-training strategies, we evaluate the intrinsic language modeling performance of our models using **perplexity** [82]. Perplexity is a widely used metric that quantifies how well a language model predicts a sequence of words; lower values indicate better generalization and more confident predictions of unseen text. The perplexity (ppl) of a model *θ* on a test set $\mathcal{W}$ is defined as the inverse probability that *θ* assigns to $\mathcal{W}$, normalized by the test set length. More formally, for a sequence of *n* words $w_{1:n} = (w_1, \ldots, w_n)$, the perplexity is given by:

1 $$\mathrm{ppl}_{\theta}(w_{1:n}) = \Pr{}_{\theta}(w_{1:n})^{-\frac{1}{n}} $$

2 $$= \sqrt[n]{\frac{1}{\Pr{}_{\theta}(w_{1:n})}}$$

We can use the chain rule of probability to express the perplexity of a sequence of words as the product of the probabilities of each word given its preceding words:

3 $$\mathrm{ppl}_{\theta}(w_{1:n}) = \sqrt[n]{\prod_{i=1}^{n} \frac{1}{\Pr{}_{\theta}(w_i | w_{1:i-1})}}$$

Note that due to the inverse relationship in Eq. 1, higher probabilities assigned to word sequences correspond to lower perplexity values. Consequently, a model with lower perplexity indicates that it is a better predictor of the given test set. Minimizing perplexity is equivalent to maximizing the probability of the test set as predicted by the LM.

### Downstream task evaluation

With minimal adjustments to its architecture, pre-trained LMs can be adapted for downstream applications by fine-tuning them on task-specific datasets. Fine-tuning involves adding task-specific layers or adaptation heads that process the model’s hidden representations. The fine-tuning process consists of continued training using labeled data from supervised datasets to adjust the weights of both the pre-trained model and the task-specific layers added on top.

To demonstrate the efficacy of our domain-adapted model in biomedical language modeling, we fine-tune and evaluate the ChristBERT models on two common biomedical downstream tasks: NER and text classification.

#### Named entity recognition

NER is used to extract relevant text spans, such as mentions of diagnoses or medications, from clinical text. We evaluated NER performance on three German biomedical corpora covering oncology and cardiology domains. **BRONCO150** [52] consists of anonymized sentences from 150 discharge summaries labeled with the categories *Medication*, *Treatment*, and *Diagnosis*. **GGPONC** [51] is a large-scale corpus derived from German oncology guidelines, containing over 200,000 named entities. There are two major versions of the corpus, from which we used the second version. For our experiments, we selected the most challenging configuration with fine-grained labels and long entity spans to ensure comparability with prior work [18]. Finally, **CARDIO:DE** [83] comprises 500 cardiovascular discharge letters annotated with six medication-related entity types. We excluded experimental sublabels from CARDIO:DE due to their low inter-annotator agreement.

#### Text classification

Text classification refers to assigning one or more labels to a document based on its content. We evaluated model performance on two multi-label German biomedical classification tasks:

**CLEF eHealth 2019** [30, 54, 55] consists of 8,385 German non-technical summarynon-technical summaries (sNTS) of planned animal studies from the AnimalTestInfo database [84]. Each summary is annotated with zero or more ICD-10 codes. We followed prior work [17] in filtering out rare classes (fewer than 25 occurrences), resulting in 5,688 documents and 230 classes. **JSynCC** [50] contains 867 synthetically generated German case reports from 10 medical textbooks, each annotated with one or more medical specialties. To address class imbalance, we again retained only frequently occurring labels, reducing the dataset to 534 documents and 6 classes, including *Trauma Surgery*, *Anesthesiology*, and *Orthopedics*.

Both datasets exhibit significant label imbalance and are treated as multi-label classification problems.

#### Evaluation metrics

We report standard classification metrics: precision, recall, and F_1_ score. Following common practice in biomedical NER and multi-label classification [85, 86], we used micro-averaged F_1_ as our primary metric to account for class imbalance and capture overall model performance.

#### Dataset preparation

**Fig. 5:**
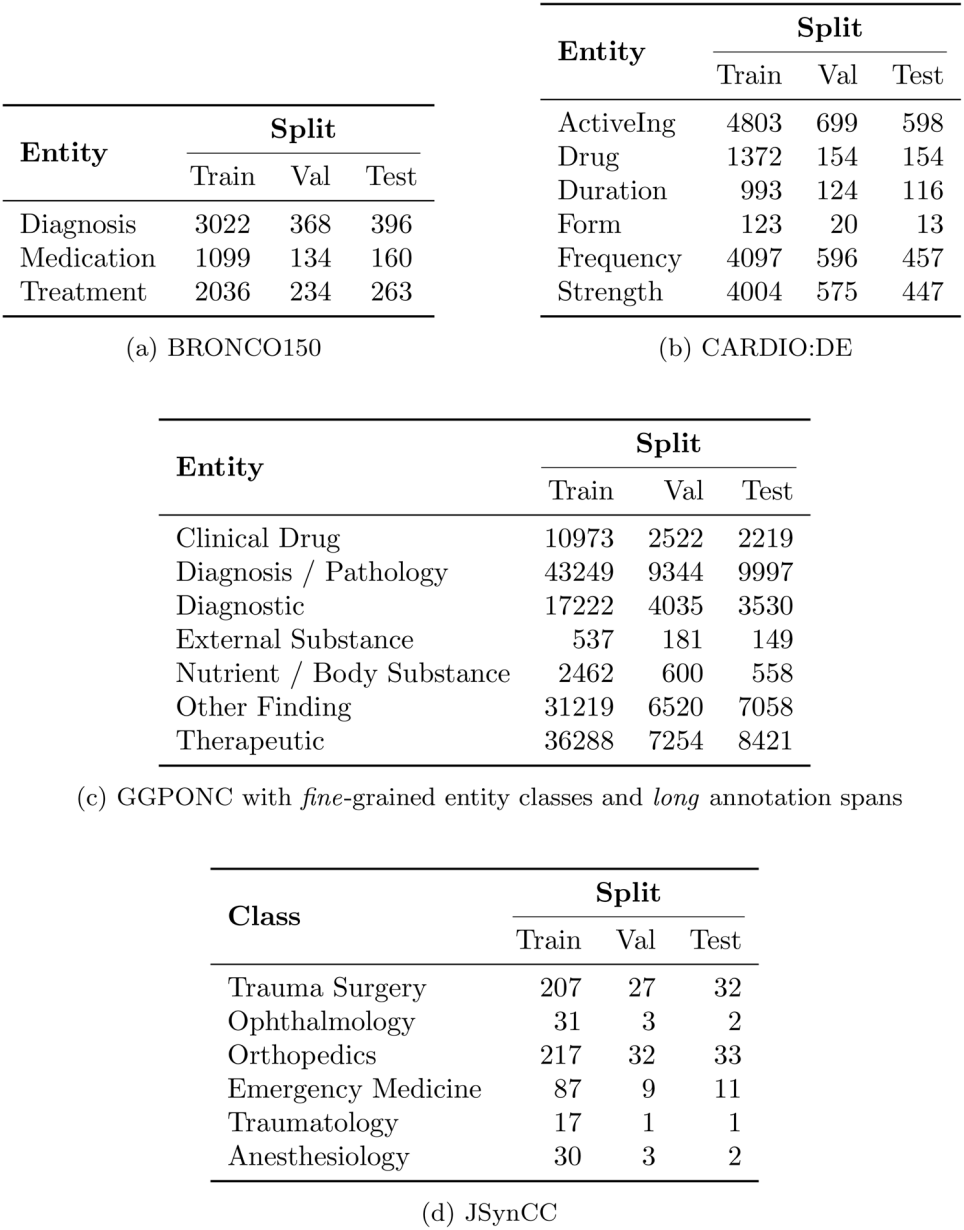
Entity and class distributions of the downstream tasks

For all NER benchmarks, we employed the BigBIO [87] library, which provides harmonized dataset schemas, standardized IOB2 entity annotations, and consistent data access tooling for biomedical NLP. For text classification tasks, we used the Hugging Face Datasets library [88], which also serves as a foundation for BigBIO. Whenever available, we preserved the official training, validation, and test splits. For datasets without predefined splits, namely BRONCO150, CARDIO:DE, and JSynCC, we applied stratified random partitioning, allocating 80, 10, and 10% of the data to training, validation, and testing, respectively. Figure 5 illustrates the label distribution across splits for each benchmark. We exclude the CLEF eHealth 2019 dataset from this figure, as it contains 230 possible classes.

#### Experimental setup

To evaluate downstream performance, we conducted fine-tuning experiments with hyperparameter optimization on each task. Specifically, we performed a grid search over batch size and learning rate, as detailed in Table 3, yielding 28 trials per task. The search space is based on the GeistBERT evaluation setup [19] and extended with additional learning rate values. Each trial used a warmup step ratio of 10% and trained for up to 30 epochs. The best model checkpoint was selected based on validation set performance. For both NER and classification tasks, we report *micro-averaged* precision, recall, and F_1_ scores on each benchmark’s test set.

**Table 3:** Hyperparameters used in the grid search for the downstream tasks

Parameter	Values
Learning Rate	$7\times10^-{5}$, $5\times10^-{5}$, $2\times10^{-5}$, $1\times10^-{5}$, $7\times10^{-6}$, $5\times10^{-6}$, $1\times10^{-6}$
Batch Size	16, 32, 48, 64

Unlike perplexity (see Section "Language modeling evaluation"), which evaluates intrinsic language modeling ability, downstream task performance enables direct comparison of model efficacy across architectures and domains. To benchmark the ChristBERT models, we selected four state-of-the-art (SOTA) German Transformer-based language models-two domain-specific and two general-purpose baselines (Table 4). All model-task combinations underwent identical hyperparameter tuning and evaluation procedures for fair comparison. A brief overview of each baseline is provided below; additional architectural details are provided in Section "Related work" of the Supplementary Material.

**Table 4:** Architecture, domain, and corpus size of evaluated models. For ChristBERT, BioGottBERT and GeistBERT, corpus size indicates the size of the initial + continuous pre-training corpus

Model	Type	Domain	Corpus
			Size (GB)
ChristBERT	RoBERTa	Biomedical	1,482 + 13.5
ChristBERT_scratch_	RoBERTa	Biomedical	13.5
ChristBERT_BPE_	RoBERTa	Biomedical	13.5
medBERT.de [18]	BERT	Biomedical	10.3
BioGottBERT [17]	RoBERTa	Biomedical	145 + 0.8
GeistBERT [19]	RoBERTa	General	145 + 1,337
GeBERTa [58]	DeBERTa	General	167

*medBERT.de* is based on the BERT [8] base architecture and is specialized for the German medical domain. Similar to ChristBERT_BPE_, it was trained from scratch with a custom domain-specific vocabulary on a large and diverse 10.3 GB corpus, comprising 4.7 million German medical documents from eleven different sources, including articles from the German health web, scientific texts, medical books, and real-world clinical data such as electronic health records and radiology reports from Charité University Hospital. This substantial dataset translated into SOTA performance on various medical benchmarks, particularly for longer and more complex texts, such as NER and ICD-10 chapter classification from radiology discharge summaries and surgical reports.

*BioGottBERT* is a domain-adapted variant of the unfiltered base version of GottBERT [36], a RoBERTa-based model trained on the German portion of the OSCAR corpus [38] (145 GB of general text). Similar to ChristBERT, BioGottBERT was *continuously pre-trained* on 809 MB of biomedical German texts, specifically from Wikipedia, scientific abstracts and drug leaflets. Despite the small biomedical corpus, BioGottBERT demonstrated notable improvements over its general-domain counterpart on a variety of medical NLP tasks, including NER and classification problems. Our benchmark selection closely follows that of BioGottBERT. The shared tokenizer enables direct comparability between ChristBERT and BioGottBERT across all tasks.

*GeistBERT* is a general-domain German language model based on the RoBERTa base architecture. It follows a *continued pre-training* approach, initializing from the best checkpoint of filtered GottBERT [36] (94,530 steps), and extending it with 100,000 further training steps using WWM. The training corpus spans 1.3 TB of partially deduplicated German data, including crawled web text and publicly accessible legal documents [89, 90]. GeistBERT achieves SOTA results across NER, classification, and natural language inference tasks, outperforming even larger models. GeistBERT serves as the general-domain reference for assessing the impact of domain-specific pre-training.

*GeBERTa* is another general-domain model that employs the DeBERTa [91] base architecture, featuring *disentangled attention* for improved contextual representation. It was trained on 167 GB of heterogeneous German data, including formal, informal, legal, medical, and literary text. GeBERTa has been evaluated on general and medical NER, sentiment analysis, hate speech detection, and question answering tasks. As a non-RoBERTa baseline, it allows comparison of architectural effects and cross-domain training data on downstream performance.

#### Implementation details

All fine-tuning experiments were conducted using the Hugging Face Transformers [92] Python library and the Neural Network Intelligence [93] framework for hyperparameter tuning. The choice of libraries was reinforced by their native support for the dataset implementations [87, 88]. For classification, model inputs were tokenized with truncation to and padding up to the maximum length of 512 tokens. In the case of NER, longer sequences were split into one or more sequences of 64 tokens except for GGPONC, whose sequences were split into 128 tokens. These values correspond to the 95th percentile of the sequence lengths across each dataset’s training, validation, and test splits. The evaluation metrics were computed using the seqeval [94] Python library for NER and the sklearn [95] Python library for classification. In seqeval, *strict* evaluation mode was applied, measuring both the correctness of the entity boundary and the entity class. All experiments were conducted on consumer-grade hardware, specifically an NVIDIA RTX 3090 GPU with 24 GB VRAM, ensuring reasonable training times for practitioners. The total computation time for all experiments encompassing all 35 model-dataset combinations, amounted to approximately 6.74 days (refer to Table S3 in the Supplementary Material).

## Results

### Pre-training performance

**Fig. 6:**
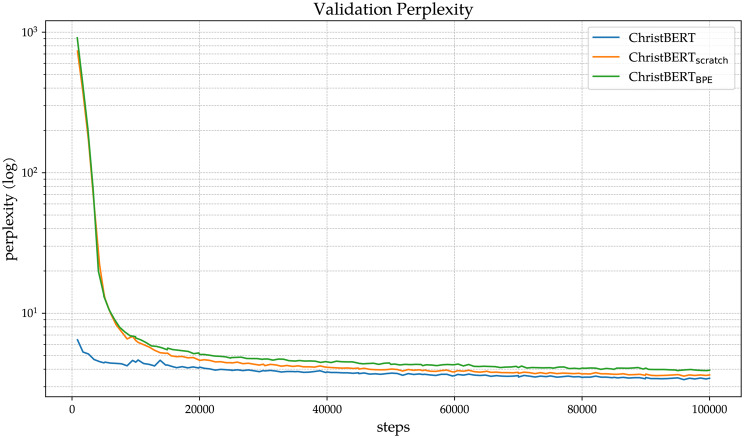
Perplexity during pre-training of ChristBERT models. Perplexity is shown in log scale for every optimization step and evaluated on the validation split of the pre-training corpus

Figure 6 plots the perplexity of the three ChristBERT models during pre-training over 100,000 training steps. Perplexity was evaluated on a held-out validation set of 3,000 randomly chosen documents from our pre-training corpus. We observe that the different domain adaptation strategies are reflected in the perplexity trajectories in terms of initial perplexity, rate of decline and convergence behavior.

#### Initial perplexity and rate of decline

Initially, the two ChristBERT variants pre-trained from scratch exhibit high perplexity values of *57434.5* and *56343.3*, while the continuously pre-trained ChristBERT starts with a lower perplexity of *12.64*. The lower perplexity directly results from the model being initialized with the weights of GeistBERT, demonstrating the effectiveness of transfer learning. As pre-training progresses, perplexity decreases steeply during the first 10,000 steps for ChristBERT_scratch_ and ChristBERT_BPE_, with the rate of perplexity reduction following a non-linear pattern across all variants. The steepest reduction occurs within the first 5,000 steps, where we observe perplexity values dropping approximately two orders of magnitude from ${\sim} 10^3$ to ${\sim} 10^1$. The observed perplexity curve can be attributed to the learning rate schedule, in which the learning rate is linearly increased for 10,000 iterations to its maximum. After this warmup phase, the perplexity trajectory flattens considerably between steps 10,000 and 40,000. Model perplexity continues to decrease but at a substantially slower rate, which is due to the learning rate following a polynomial decay to zero after the first 10,000 steps.

#### Convergence behavior

The continuously pre-trained ChristBERT model converges the fastest, stabilizing at around a perplexity of 3–4 by 10,000 steps and maintaining the lowest perplexity throughout pre-training. Additionally, ChristBERT consistently achieved lower perplexity than both BPE and Scratch variants, with perplexity values 30–50% lower during the middle stages of pre-training. Despite this, after around 40,000–50,000 iterations, all models reach a relatively stable perplexity level between 2 and 4. Diminishing returns are observed after 60,000 steps, suggesting extended pre-training offers minimal improvements and convergence is achieved. Moreover, we observed divergence in some pre-training runs, particularly due to high learning rates where model parameters were updated too aggressively. This divergence manifested as an abrupt loss of convergence, characterized by a dramatic increase in perplexity that would have persisted for the remainder of training, as shown in Fig. S1 in the Supplementary Material. To mitigate divergence, we found that lowering the learning rate was effective in stabilizing pre-training. This was necessary for ChristBERT_scratch_ and ChristBERT_BPE_, where we reduced the maximum learning rate from $7\times10^{-4}$ to $6\times10^{-4}$, while with the GeistBERT initialization it was possible to use a higher peak learning rate.

ChristBERT_BPE_ consistently demonstrates the highest perplexity values among the three variants, particularly during the middle phase of pre-training. This effect likely stems from its custom byte-pair encoding vocabulary, during which it learns different language representations from scratch. As discussed in Section "Language modeling evaluation", perplexity comparisons are most meaningful between models sharing the same tokenizer; therefore this difference does not necessarily indicate inferior model quality. Moreover, an improvement in an intrinsic measure such as perplexity does not necessarily correlate with enhanced performance in extrinsic measures such as practical downstream language tasks. Nevertheless, perplexity remains a useful proxy for estimating a model’s generalization capacity and its potential effectiveness on downstream tasks.

### Fine-tuning performance

#### Named entity recognition

Table 5 shows the performance results of medical named entity recognition on the BRONCO150, CARDIO:DE, and GGPONC datasets. Detailed results for each entity type in the respective dataset are reported in Table S7–S9 in the Supplementary Material. The ChristBERT models consistently outperform the baseline models across all datasets, establishing a new state-of-the-art for German biomedical NER.

**Table 5:** Overview of micro averaged precision (prec.), recall (rec.) and F_1_ scores on the NER tasks. All results are shown in percent and assess each model’s best fine-tuned performance on each downstream task’s test set. The best model was selected out of 28 runs based on its validation set performance. Best score in bold and second best underlined

Model	BRONCO150			CARDIO:DE			GGPONC		
	Prec.	Rec.	F_1_	Prec.	Rec.	F_1_	Prec.	Rec.	F_1_
ChristBERT	81.42	81.77	81.87	85.58	89.65	87.57	75.65	**79.83**	**77.69**
ChristBERT_scratch_	81.87	82.32	82.09	88.38	89.89	89.13	76.54	77.56	77.05
ChristBERT_BPE_	**85.71**	**83.78**	**84.74**	89.50	**91.31**	**90.40**	**76.59**	77.42	77.00
medBERT.de	78.67	79.58	79.12	87.66	90.02	88.83	73.89	75.78	74.73
BioGottBERT	76.96	78.45	77.70	88.37	90.74	89.54	75.24	75.40	75.32
GeistBERT	75.65	79.83	77.69	85.58	89.65	87.57	74.57	75.36	74.96
GeBERTa	78.67	79.58	79.12	**90.51**	90.23	90.37	75.96	76.93	76.45

On the BRONCO150 dataset, ChristBERT_BPE_ achieves the highest precision (85.71%), recall (82.32%) and F_1_ score (84.74%), marking a substantial improvement over both specialized medical models and general language models. ChristBERT_scratch_ places second with an F_1_ of 83.33%, followed closely by ChristBERT with 81.87%. The performance delta between ChristBERT variants and other models is particularly evident when comparing against the general language models. For instance, the F_1_ score of ChristBERT_BPE_ with 84.74% represents a 5.62% point improvement over GeistBERT (77.69%) and a 5.08% point improvement over GeBERTa (79.12%). This significant performance gap underscores the value of domain-specific pre-training for NER in medical texts.

The performance on the CARDIO:DE dataset presents a different pattern of results. Here, all compared models showed more similar performances, with ChristBERT_BPE_ performing the best, followed closely by GeBERTa, which leads among the baselines and demonstrates comparable NER efficacy. Both mentioned models achieve high F_1_ scores of 90.40% and 90.37%, respectively, differing in precision and recall. This dataset highlights the potential for general language models to perform competitively in certain medical subdomains when trained appropriately.

The GGPONC dataset presents the most challenging evaluation scenario with eight fine-grained semantic classes and long entity spans across a large corpus of oncology documentation. On this complex dataset, ChristBERT models again demonstrate superior performance compared to the baseline models, with ChristBERT achieving the highest recall (79.83%), while ChristBERT_BPE_ attains the highest precision (76.59%). Here, ChristBERT_BPE_ and ChristBERT_scratch_ match each other’s precision, recall and F_1_ scores. The performance advantage of our pre-training corpus on GGPONC is particularly noteworthy given the complexity of this dataset. With an F_1_ of 77.69%, ChristBERT is the best performing NER model and outperforms the next best non-ChristBERT model GeBERTa at 76.45% by 1.24% points. The demonstrated advantage in the most complex dataset suggests that the domain-specific pre-training of ChristBERT models enables more effective learning of the nuanced entity boundaries and semantic distinctions required for fine-grained medical entity recognition.

#### Text classification

Table 6 presents the classification results for each model on the CLEF and JSynCC classification datasets. Detailed results for each topic category in JSynCC can be found in Table S9 in the Supplementary Material. We omit a separate per-class drill-down for the CLEF dataset as it contains over 230 classes. As such, the CLEF benchmark poses the more challenging multi-label classification task, while JSynCC only requires assigning labels from six medical categories.

**Table 6:** Overview of micro averaged precision (prec.), recall (rec.) and F_1_ scores on the classification tasks. All results are shown in percent and assess each model’s best fine-tuned performance on each downstream task’s test set. The best model was selected out of 28 runs based on its validation set performance. Best score in bold and second best underlined

Model	CLEF			JSynCC		
	Prec.	Rec.	F_1_	Prec.	Rec.	F_1_
ChristBERT	78.12	75.34	76.03	89.01	**100**	94.19
ChristBERT_scratch_	**93.68**	85.17	89.22	91.86	97.53	**94.61**
ChristBERT_BPE_	88.22	88.35	88.28	89.53	95.06	92.22
medBERT.de	89.21	87.59	88.40	91.25	90.12	90.68
BioGottBERT	88.30	87.90	88.10	88.89	98.77	93.57
GeistBERT	90.43	72.92	80.74	**92.59**	92.59	92.59
GeBERTa	88.91	**89.71**	**89.31**	**92.59**	92.59	92.59

On the CLEF dataset, GeBERTa achieves the highest F_1_ score at 89.31%, driven by its superior recall at 89.71%. Nonetheless, ChristBERT_scratch_ demonstrates the highest precision (93.68%), indicating that it is more effective at minimizing false positives. However, its recall (85.17%) is lower than GeBERTa’s, resulting in a lower overall F_1_ at 89.22%. To our surprise, we observe that both general and domain-specific models perform similarly on this dataset. Notably, the continuously pre-trained ChristBERT variant shows the lowest overall performance among the evaluated models on this dataset with an F_1_ of 76.03%. Its performance differs by 4.61% points from the next best model GeistBERT (80.64%), its general domain counterpart. This suggests that the continuous pre-training approach may not be as effective for complex multi-label classification problems, particularly when compared to the other ChristBERT variants, which were pre-trained with the same corpus but with different initialization strategies.

It should be noted that GeBERTa included CLEF data in its pre-training corpus, meaning it had already seen this data before evaluation. This might explain its exceptionally high performance compared to other models and should be considered when interpreting these results. Even so, medBERT.de achieves strong performance with an F_1_ score of 88.40%, demonstrating that domain adaptation across different medical subdomains supports the processing of specialized terminology and concepts in animal experiment documentation.

On the JSynCC dataset, the majority of ChristBERT models considerably outperform the baseline models, with ChristBERT_scratch_ achieving the highest F_1_ score of 94.61%, closely followed by ChristBERT at 94.19% and a shared third place between GeistBERT and GeBERTa at 92.59%. A particularly striking observation is the perfect recall (100%) of ChristBERT on the JSynCC dataset, indicating that it identifies all relevant specialty classifications across the test documents. However, its precision (89.01%) is lower than other models, resulting in an F_1_ of 94.19%. This pattern suggests that ChristBERT may be over-predicting certain class labels, but its comprehensive coverage ensures no relevant classifications are missed, a characteristic that could be valuable in clinical applications where missing a relevant specialty category might have significant consequences.

The performance clustering on JSynCC is notably tight, with all models achieving F_1_ scores between 92.59% and 94.61%. Notably, BioGottBERT achieves the second-highest overall performance on JSynCC with an F_1_ of 93.57% and recall of 98.77%. This suggests that the synthetic nature of this corpus may present more standardized linguistic patterns that various model architectures can effectively learn during fine-tuning. Furthermore, while ChristBERT_BPE_ has consistently shown the best performance in NER tasks, it does not rank among the top models on all classification benchmarks. This indicates that the BPE vocabulary may not be as effective for text classification tasks, where the model’s ability to generalize across different contexts and semantic meanings is crucial.

#### Cross-model analysis and domain specialization effects

Among the ChristBERT variants, ChristBERT_BPE_ consistently demonstrates strong performance across all NER datasets, achieving the highest or second-highest F_1_ scores in each experiment. This suggests that the custom BPE vocabulary approach may offer advantages for handling the morphological complexity and specialized vocabulary found in German medical texts. Despite its seemingly weaker performance during pre-training as indicated by higher perplexity values, its downstream performance confirms that pre-training metrics do not necessarily translate into task-specific effectiveness.

ChristBERT_scratch_ also performs competitively across NER datasets, indicating that domain-specific training from initialization can be effective without leveraging transfer learning from general domain pre-training. The continuously pre-trained ChristBERT model shows particular strength in the GGPONC dataset, suggesting it may have advantages for handling complex, fine-grained entity recognition tasks.

The comparison between specialized medical models (ChristBERT variants, medBERT.de, BioGottBERT) and general language models (GeistBERT, GeBERTa) reveals distinct performance behavior in medical NER. In BRONCO150 and GGPONC, domain-specific models generally outperform general models, confirming the value of specialized pre-training for oncology text. However, in CARDIO:DE, GeBERTa achieves the highest F_1_, suggesting that general language models can be competitive in certain medical subdomains when trained on heterogeneous and cross-domain data. Notably, 8% of GeBERTa’s pre-training data consisted of medical texts.

This variability illustrates that domain specificity presents different advantages depending on the particular medical subdomain and entity types being targeted. The general language models appear more competitive on CARDIO:DE, possibly due to differences in writing style, terminology standardization, or entity class definitions between cardiovascular and oncology domains. Interestingly, we observe GeistBERT exhibiting equivalent performance to the domain-adapted model BioGottBERT. We attribute this mainly to the relatively small size of BioGottBERT’s biomedical training corpus (0.8 GB), highlighting the importance of corpus size in achieving effective domain adaptation.

An analysis of precision and recall values reveals different optimization patterns across models. ChristBERT_BPE_ tends to favor precision over recall in BRONCO150 and GGPONC, while achieving high values in both metrics for CARDIO:DE. In contrast, the continuously pre-trained ChristBERT shows stronger recall performance, particularly in GGPONC. These trade-offs have important implications for clinical applications, where the relative importance of precision versus recall may vary based on the specific use case.

For the classification tasks, a complementary pattern emerges. While ChristBERT_BPE_ dominated in NER, it was outperformed by ChristBERT_scratch_ and the baseline GeBERTa on both CLEF and JSynCC. This suggests that the advantages of byte-pair encoding may not generalize equally across all task types. In contrast, ChristBERT_scratch_ delivered consistently strong results in both precision and recall, particularly excelling on JSynCC, which implies that full pre-training on domain-specific corpora enables robust feature representations for document-level tasks.

The continuously pre-trained ChristBERT variant showed the weakest classification performance, likely due to residual biases from general-domain pre-training interfering with adaptation to complex, multi-label classification setups like CLEF. Interestingly, despite its poor performance on CLEF, this variant achieved perfect recall on JSynCC, underscoring that continued pre-training can support comprehensive label coverage but may lead to over-prediction and reduced precision.

## Discussion

### General findings

In this study, we systematically explored three complementary strategies for domain adaptation of German biomedical language models: continued pre-training from a general-domain model (ChristBERT), pre-training from scratch (ChristBERT_scratch_), and vocabulary adaptation via domain-specific subword tokenization (ChristBERT_BPE_). All models were pre-trained on a newly curated 13.5 GB biomedical corpus and evaluated on downstream biomedical tasks, including NER and text classification. Our experiments reveal several principal findings regarding the three investigated domain adaptation strategies.

First, continued pre-training proved particularly effective in terms of efficiency. ChristBERT achieved the lowest perplexity and converged fastest, underscoring the benefits of leveraging general-domain knowledge. This advantage, however, did not always translate into downstream superiority for initializing biomedical models. Instead, its performance varied among downstream tasks: While ChristBERT excelled in NER, particularly on GGPONC, it ranked lowest on complex classification tasks such as CLEF, indicating that inherited general-domain priors may not always be beneficial for complex classification tasks.

Second, pre-training from scratch led to robust and often superior downstream performance. ChristBERT_scratch_ achieved top results on text classification tasks, particularly JSynCC, where it attained the highest F_1_ score. This suggests that domain-exclusive representations learned from scratch may offer advantages in classification scenarios requiring broader semantic coverage and contextual generalization.

Third, domain-specific vocabulary adaptation (ChristBERT_BPE_) yielded the strongest performance for entity-centric tasks. Despite higher perplexity during pre-training, this variant excelled in NER tasks across all datasets, achieving state-of-the-art results on BRONCO150 and CARDIO:DE. However, its performance in classification tasks was less competitive, indicating that the benefits of domain-optimized tokenization are most pronounced in tasks sensitive to terminological precision and morphological complexity.

Finally, comparisons to general-purpose language models highlighted the importance of domain adaptation. While general models such as GeistBERT and GeBERTa remained competitive on certain datasets like CARDIO:DE and CLEF, they were consistently outperformed by the ChristBERT variants on more specialized or complex biomedical tasks. Furthermore, smaller-scale domain adaptation efforts (e.g., BioGottBERT) could not match the performance gains achieved through our larger corpus and comprehensive pre-training strategies.

In summary, our findings emphasize that no single adaptation strategy universally outperforms the others. Continued pre-training offers rapid convergence and strong generalization. From-scratch pre-training provides robust performance for classification, while additional domain-specific vocabulary is most beneficial for specialized tasks like NER. Our results highlight that the suitability of domain-specific tokenization strategies, such as a custom BPE vocabulary, is highly task-dependent. This suggests that domain-specific BPE tokenization is especially beneficial for entity recognition, where accurate boundary detection and handling of rare terms are critical. In contrast, classification tasks often rely more on the model’s ability to generalize over broader semantic and syntactic patterns rather than fine-grained tokenization. Thus, in such contexts, the rigid subword splits introduced by domain-specific BPE may offer less benefit, or even introduce unnecessary complexity. These observations emphasize the importance of aligning vocabulary adaptation strategies not only with the domain but also with the linguistic properties and demands of the target task.

### Findings in the context of prior work

Our findings align well with and extend previous work on domain-adaptive pre-training. The study [96] demonstrated that continued pre-training yields significant gains for domain-specific tasks, especially when the target domain is distant from the original pre-training corpus. Our results confirm this for German biomedical NLP: continued pre-training (ChristBERT) led to rapid convergence and strong performance in complex NER tasks like GGPONC. Furthermore, previous findings from [97] suggest that training from scratch can be competitive with, or even outperform, continued pre-training on biomedical classification tasks. Our ChristBERT_scratch_ model demonstrated this by excelling on both the JSynCC and CLEF classification benchmarks. In their experiments, the authors of [97] also observed that medical-specific vocabularies lead to performance gains in downstream domain tasks. Our results mirror these prior observations, with ChristBERT_BPE_ achieving top results in NER, reinforcing the idea that domain-aligned vocabulary improves handling of specialized terminology.

Inspired by [20], we translated English medical texts into German to address the scarcity of native-language biomedical corpora. This strategy proved effective in terms of downstream task performance compared to medBERT.de [18], which relied exclusively on original German data. GeBERTa [58], which also leveraged translated medical texts, achieved similarly strong results, particularly in classification scenarios. Notably, even general-purpose models performed competitively on classification tasks, underscoring that large-scale general-domain pre-training enriched with some biomedical content remains a viable approach for such tasks. Nevertheless, our findings support the approach of translation-based corpus construction, especially for tasks like biomedical NER, where domain-specific nuances and terminology require targeted representation learning and original German resources remain limited.

While implementing the translation strategy for MIMIC-IV with LLaMA 3.1 and Pubmed Central with NLLB 200, similar to [58] we also observed that the quality of the machine-translated data was sensitive to translation settings. In particular with NLLB 200, we noticed that larger context sizes and sequence lengths frequently resulted in degraded translation quality. Phenomena such as stuttering and incoherent phrase repetition became evident, especially in complex biomedical sentences. This degradation can stem from several factors inherent to current translation models and LLMs. For instance, generic LLMs, if configured incorrectly during inference (e.g. insufficient context window sizes), may fail to attend to the entire input sequence, effectively *forgetting* earlier parts of the input and producing incomplete or nonsensical translations. Likewise, many dedicated translation models are trained on sentences. Consequently, their ability to handle longer sequences degrades, as the positional embeddings beyond the trained length are less reliable, leading to instability and errors in translation [22]. To ensure the reliability of the translated corpus, we therefore opted for a context size of 384 tokens for NLLB 200, which offered a favorable balance between translation throughput and linguistic accuracy, mitigating some of these input length-related issues.

### Limitations and future work

While this study provides valuable insights into domain adaptation strategies for German biomedical language models, several limitations remain and point to promising directions for future research.

Our investigation was limited to the RoBERTa architecture, following the design path of GeistBERT [19] and GottBERT [36]. Although this ensured comparability, alternative Transformer architectures, including the recently introduced ModernBERT [98] and its published German variant ModernGBERT [99], represent interesting directions for future research. However, recent findings suggest that architectural novelty does not necessarily translate into improved downstream performance compared to well-trained RoBERTa-based encoders [100–103]. Consequently, whether such architectures provide tangible benefits in the German biomedical setting remains an open empirical question. RoBERTa’s maximum input size might become particularly constraining in biomedical contexts, where clinical documents such as patient records or scientific articles often involve extended contexts. Future work should explore long-context Transformers. Architectures such as Longformer [104] and Nyströmformer [105] could offer significant advantages in tasks requiring document-level understanding or the resolution of complex cross-sentence dependencies [106].

Nevertheless, provided that sufficient training data are available, RoBERTa-based encoder models have been shown to be highly efficient [100, 103] and benefit from standardized and comparatively straightforward pre-training pipelines [81]. Since the present study focuses on encoder architectures, it may be interesting to investigate whether recent decoder-only models, such as Llama 3.1 [107] or Qwen 3 [108], exhibit similar behavior in the German biomedical domain. However, Zhang et al. [109] recently suggested that encoder models may still outperform decoder models on certain downstream tasks despite their substantially smaller size and computational demands.

Additionally, future work should extend the evaluation of ChristBERT to additional German biomedical benchmarks as they become available. Resources developed within initiatives such as GeMTeX [110–112] may provide opportunities to assess model performance beyond named entity recognition and text classification. In particular, tasks such as information extraction, relation extraction, and question answering represent promising directions for future evaluation. However, to the best of our knowledge, no publicly available German biomedical benchmark datasets covering these tasks were available at the time this study was conducted.

Furthermore, our findings indicate that training from scratch can be most effective under certain conditions. This discrepancy highlights the need for further investigation into the factors that influence the effectiveness of training from scratch versus continued pre-training. Future work should focus on exploring the specific scenarios and downstream tasks where training from scratch might outperform continued pre-training. It would be valuable to conduct a more detailed analysis of the trade-offs between computational resources, training time, and performance gains. Understanding these dynamics could provide insights into optimizing model training strategies for various applications. Similarly, while our models build directly on GeistBERT and GottBERT in terms of tokenizer design and vocabulary size, these decisions were not revisited for the biomedical domain. Given the distinct lexical properties of medical language, alternative vocabulary sizes or tokenization schemes might further optimize model performance.

Moreover, the range of biomedical benchmarks used, while diverse, does not fully reflect the variety of clinical language processing needs. Tasks involving decision support, complex narratives, and clinical reasoning were underrepresented, which will hopefully change in the near future with the release of the German Medical Text Corpus Project [113]. Addressing these gaps is important, especially in light of models like medBERT.de [18], which explicitly targeted such scenarios. In addition, subdomains such as radiology, psychiatry, and primary care were not systematically explored, limiting our conclusions about generalizability.

Our corpus design also presents limitations. While our approach exclusively used biomedical data, models like GeBERTa [58] have demonstrated that mixed-domain corpora can enhance generalization, particularly for tasks that bridge specialized and general language. Investigating mixed corpus strategies within the same RoBERTa architecture could therefore provide deeper insights into optimal corpus design for domain-adaptive pre-training. Further considerations result from performing translation to augment the pre-training corpus. Although translation enabled the creation of a large biomedical corpus, the quality of this synthetic data was not manually verified by healthcare professionals and, as discussed, was sensitive to context size, with larger sizes impairing coherence. Consequently, it remains unclear to what extent translation artifacts affected downstream performance. Systematic analyses, including expert-driven manual reviews and targeted error analyses, could provide valuable insights into the role of translation quality in biomedical language modeling, support the development of mitigation strategies, and improve our understanding of translation model behavior in this setting. Likewise, we did not systematically analyze the individual contributions of the different data sources within our corpus; it remains unclear to what extent the translated data specifically improved performance compared to relying solely on the original German sources. Additionally, de-identified datasets, i.e. MIMIC-IV, contain artifacts such as anonymization masks, which are not typically found in natural prose, potentially affecting performance on other types of text. As a byproduct of the translation effort, we have obtained a large bilingual corpus of clinical texts (MIMIC-IV Notes) and biomedical literature (PMC). This corpus could be used to fine-tune German-English translation models for the biomedical domain, supporting both direct clinical applications and future corpus creation.

Lastly, one should be cautious when interpreting results in cases of potential data leakage. In the case of GeBERTa and medBERT.de, the pre-training corpus included GGPONC, which is also part of our NER evaluation. However, medBERT.de performed worse than several models without similar data leakage, suggesting that this exposure did not translate into a measurable advantage in case of NER. This underlines the importance of careful dataset curation and transparency when reporting benchmark results, while also showing that plain-text overlap alone does not guarantee performance gains. It remains unclear whether, and to what extent, such data leakage impacts downstream performance, especially since GGPONC is relatively small compared to the full pre-training corpora. Determining their exact influence would require dedicated experiments, but we highlight the potential of such effects for further investigation. Although care was taken to exclude downstream evaluation corpora from the ChristBERT pre-training corpus, we cannot completely rule out the possibility that isolated excerpts from these resources were indirectly included through citations or quotations contained in publicly available documents, such as doctoral or postdoctoral dissertations. However, no evaluation datasets were intentionally incorporated during corpus construction. Given the heterogeneous nature and scale of the pre-training corpus, comprising more than 1.1 million documents and 13 GB of text, we consider substantial overlap with the evaluation benchmarks to be unlikely.

## Conclusion

This study systematically explored domain adaptation strategies for German biomedical language models: continued pre-training from a general-domain model, training from scratch on biomedical data, and adapting the tokenizer with domain-specific BPE. Central to this effort was the creation of a large-scale pre-training corpus, enriched through translation-based data augmentation to address the scarcity of German clinical text.

Three models were trained using these strategies and benchmarked against existing general and medical German models. Evaluations included intrinsic perplexity and extrinsic performance across five NER and classification datasets. The ChristBERT models achieved state-of-the-art results in 4 of 5 tasks of our setup, though no single strategy consistently outperformed the others. Continued pre-training proved efficient and strong on certain NER tasks; training from scratch excelled in classification; BPE adaptation offered nuanced gains, particularly for specialized terminology. Based on our evaluations, the optimal adaptation strategy depends on task requirements and resource constraints.

This work contributes state-of-the-art German biomedical language models and provides valuable insights into domain adaptation strategies, paving the way for future advancements in clinical text processing and mining. All models and selected supporting resources are publicly released to support continued research and application.

## Electronic supplementary material

Below is the link to the electronic supplementary material.

Supplementary Material 1

## Data Availability

The downstream evaluation datasets used in this study are listed below.Persistent identifiers are provided where available. • **BRONCO150: **https://www2.informatik.hu-berlin.de/~leser/bronco/index.html. • **GGPONC 2.0:** https://doi.org/10.5281/zenodo.12518458. • **CARDIO:DE, version 1.1.2:** https://doi.org/10.11588/data/AFYQDY. • **CLEF eHealth 2019 Task 1** comprises the Non-technical Summaries (NTS) of Animal Experiments Indexed with ICD-10 Codes (version 1.0) and the corresponding official test set, both published in the Open Agrar Repository under https://doi.org/10.17590/20190118-134645-0 and https://doi.org/10.17590/20190506-101759, respectively. • **Jena Synthetic Clinical Case Corpus (JSynCC), version 1.1:** available from the JULIELab repository on GitHub at https://github.com/JULIELab/jsyncc/tree/1.1. Further details on the downstream datasets and their preparation are provided in Section "Downstream task evaluation". The pre-training corpus consists of publicly available and licensed biomedical sources, including open-access medical literature, de-identified clinical notes, and curated web data. Redistribution of the compiled corpus is restricted because some source collections are subject to licensing and access conditions. Further details on its composition, sources, and preprocessing are provided in Section "Corpus creation" and Table 1.

## Abbreviation

AI: Artificial intelligence
BERT: Bidirectional Encoder Representations from Transformers
BPE: Byte-Pair Encoding
BRONCO150: Berlin–Tubingen Oncology Corpus
CLEF: Conference and Labs of the Evaluation Forum
DOI: Digital Object Identifier
GGPONC: German Guideline Program in Oncology NLP Corpus
i2b2: Informatics for Integrating Biology and the Bedside
ICD-10: International Classification of Diseases and Related Health Problems
JATS: Journal Article Tag Suite
JSynCC: Jena Synthetic Clinical Case Corpus
mBERT: multilingual BERT
MIMIC-IV: Medical Information Mart for Intensive Care IV
n2c2: National NLP Clinical Challenges
NCBI: National Center for Biotechnology Information
NER: Named Entity Recognition
NLM: National Library of Medicine
NLP: Natural Language Processing
NTS: Non-technical summary
OSCAR: Open Super-large Crawled ALMAnaCH coRpus
PMC: PubMed Central
ppl: perplexity
RNN: recurrent neural network
RoBERTa: Robustly optimized BERT Pretraining approach
sGHW: sampled German Health Web
SOTA: state-of-the-art
WWM: Whole Word Masking

## Materials availability

The pre-trained ChristBERT models are publicly available at https://huggingface.co/ChristBERT. Fairseq checkpoints can be provided upon request.

## Code availability

Code and software resources supporting this study are publicly available under the MIT License in the ChristBERT group on GitLab: https://gitlab.com/christbert.
