# Supplementary material for: The word and the way: strategies for domain-specific BERT pre-training in German medical NLP
Source: BMC Med Inform Decis Mak. 2026 Aug 6;26:296. doi: 10.1186/s12911-026-03728-2 (PMC13449853; doi:10.1186/s12911-026-03728-2)
Supplement: Supplementary file 1 [file 12911_2026_3728_MOESM1_ESM.pdf]

Supplementary Material

1 Perplexity

Fig. S1 illustrates the training instability observed during the diverged pre-training of ChristBERT$_{scratch}$. The plot shows perplexity on the validation split of the pre-training corpus across optimization steps. A sharp increase in perplexity is visible around step 12,500, indicating a failure to converge.

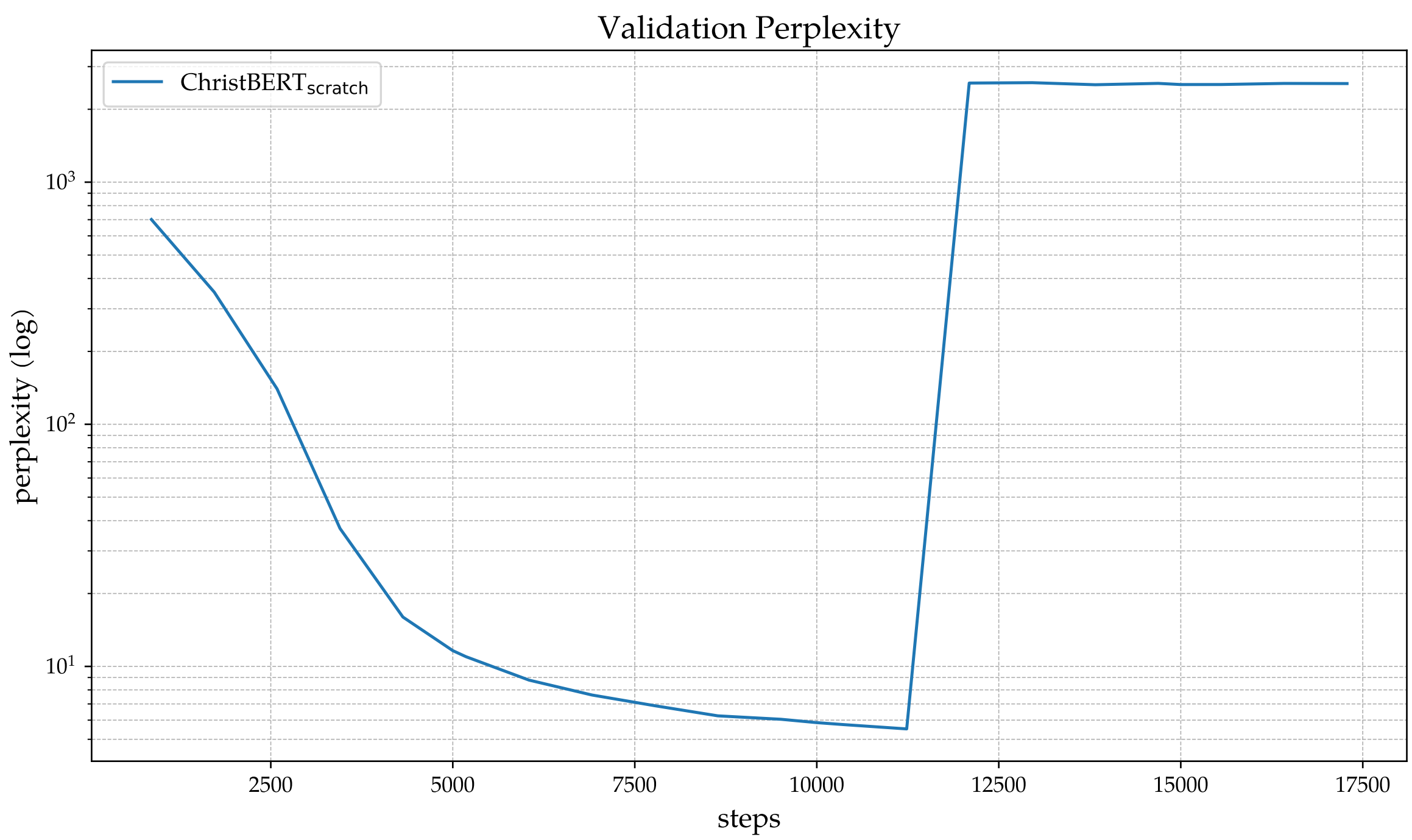

Fig. S1: Perplexity during diverged pre-training of ChristBERT$_{scratch}$. Perplexity is shown in log scale for every optimization step and evaluated on the validation split of the pre-training corpus. The plot illustrates a sharp increase in perplexity around the 12,500th step, indicating model instability and failure to converge.

2 Model Properties

Table S1 summarizes the vocabulary size and number of parameters for each evaluated model. While this table focuses on model size, other architectural differences are not shown.

Model	Vocab Size	# Parameters
ChristBERT	52,009	125,985,024
$\text{ChristBERT}_{\text{scratch}}$	52,009	125,985,024
$\text{ChristBERT}_{\text{BPE}}$	52,009	125,985,024
medBERT.de	30,000	109,081,344
BioGottBERT	52,009	125,985,024
GeistBERT	52,009	125,985,024
GeBERTa	50,266	138,620,928

Table S1: The vocabulary size and parameter size are shown for the evaluated models. This table does not show other design differences of the models. Values extracted using Huggingface Transformers library.

3 Timing and Hyperparameter Search Overview

The total computation time required for pre-training is detailed in Table S2. In addition, Table S3 reports the time spent on hyperparameter grid search for all downstream tasks, performed on a single NVIDIA RTX 3090 GPU. Table S4 lists the fine-tuning (FT) and inference (PT) runtimes for the final selected models, also measured on the same hardware.

The best-performing hyperparameter configurations (batch size and learning rate) for each task and model are provided in Table S5.

Model	Computation Time (DD:HH:MM)	GPUs	VRAM
ChristBERT	6:20:09	4 × A100 SXM	80 GB
$\text{ChristBERT}_{\text{scratch}}$	6:19:13	4 × A100 SXM	80 GB
$\text{ChristBERT}_{\text{BPE}}$	7:09:12	2 × H100	93 GB

Table S2: Pre-training computation time in days, hours and minutes summing up to 521 hours and 54 minutes, which are approximately 21.74 days.

Model	BRONCO150	CARDIO:DE	GGPONC	CLEF	JSynCC
ChristBERT	1:20:26	4:40:01	11:26:14	11:24:22	2:10:02
$\text{ChristBERT}_{\text{scratch}}$	1:28:28	5:14:12	10:47:27	12:16:22	2:30:59
$\text{ChristBERT}_{\text{BPE}}$	1:12:09	4:55:18	10:51:00	11:45:43	2:25:13
medBERT.de	1:57:32	4:31:53	11:25:21	12:53:27	2:13:56
BioGottBERT	1:18:15	6:01:10	10:55:55	12:50:17	2:03:28
GeistBERT	1:27:57	4:40:46	11:25:43	12:51:41	2:15:12
GeBERTa	1:57:32	7:39:51	19:22:22	29:15:23	3:09:10

Table S3: Computation time in hours, minutes and seconds spent on the hyperparameter grid search for finding the best models for each task. The grid search was performed on a single NVIDIA RTX 3090 GPU with 24 GB VRAM. The total computation time for hyperparameter optimization sums up to 161 hours and 46 minutes, which are approximately 6.74 days.

Model	BRONCO150		CARDIO:DE		GGPONC		CLEF		JSynCC	
	FT	PT	FT	PT	FT	PT	FT	PT	FT	PT
ChristBERT	03:51	0.13	18:27	1.30	30:06	5.08	26:33	0.91	03:05	0.18
$\text{ChristBERT}_{\text{scratch}}$	02:40	0.11	15:16	0.87	30:03	5.14	29:51	0.94	06:19	0.18
$\text{ChristBERT}_{\text{BPE}}$	02:03	0.13	11:09	1.01	24:53	5.45	26:32	1.08	07:16	0.39
medBERT.de	04:17	0.13	08:07	0.95	25:32	5.81	27:30	1.06	04:56	0.21
BioGottBERT	05:46	0.11	16:48	0.92	32:47	5.14	29:13	1.05	05:28	0.19
GeistBERT	04:05	0.19	15:14	0.88	29:57	4.83	29:45	0.96	04:09	0.19
GeBERTa	04:17	0.13	28:47	1.36	49:41	7.23	56:48	2.05	06:11	0.41

Table S4: Fine-tuning (FT) runtime in minutes and seconds, and prediction runtime (PT) in seconds of the best downstream task models for each task. Both were performed on one NVIDIA RTX 3090 GPU with 24 GB VRAM.

Model	BRONCO150		CARDIO:DE		GGPONC		CLEF		JSynCC	
	BS	LR	BS	LR	BS	LR	BS	LR	BS	LR
ChristBERT	48	7×10^{-5}	48	7×10^{-5}	16	7×10^{-5}	16	5×10^{-5}	48	5×10^{-5}
$\text{ChristBERT}_{\text{scratch}}$	32	5×10^{-5}	16	5×10^{-5}	16	7×10^{-5}	16	2×10^{-5}	64	5×10^{-5}
$\text{ChristBERT}_{\text{BPE}}$	32	7×10^{-5}	32	5×10^{-5}	32	7×10^{-5}	16	7×10^{-5}	16	5×10^{-6}
medBERT.de	16	5×10^{-5}	48	7×10^{-5}	32	5×10^{-5}	32	7×10^{-5}	64	2×10^{-5}
BioGottBERT	16	7×10^{-5}	16	5×10^{-5}	16	7×10^{-5}	16	7×10^{-5}	16	7×10^{-5}
GeistBERT	16	2×10^{-5}	16	5×10^{-5}	16	5×10^{-5}	16	2×10^{-5}	16	7×10^{-5}
GeBERTa	16	5×10^{-5}	16	7×10^{-5}	16	5×10^{-5}	48	7×10^{-5}	32	5×10^{-5}

Table S5: Hyperparameters of the best downstream task models for each task and pre-trained model. BS and LR denote batch size and learning rate, respectively.

4 Downstream Task Evaluation

Tables S6 through S9 present a detailed breakdown of evaluation results on the downstream tasks. For each dataset, BRONCO150 (Table S6), CARDIO:DE (Table S7), GGPONC (Table S8), and JSynCC (Table S9), the precision, recall, and F1-scores are reported for each class or entity.

All results are shown as percentages and refer to the best fine-tuned model selected based on validation set performance out of 28 grid search runs. The best results are highlighted in bold and the second-best are underlined.

**Model**	**Diagnosis**			**Medication**			**Treatment**		
	Prec.	Rec.	F_1	Prec.	Rec.	F_1	Prec.	Rec.	F_1
ChristBERT	<u>79.78</u>	**81.56**	<u>80.66</u>	85.71	81.36	83.48	81.89	83.87	82.87
ChristBERT$_{scratch}$	78.89	79.33	79.11	<u>87.50</u>	83.05	<u>85.22</u>	<u>83.59</u>	<u>86.29</u>	<u>84.92</u>
ChristBERT$_{BPE}$	**82.63**	<u>80.00</u>	**81.29**	**88.41**	84.72	**86.52**	**88.82**	**88.82**	**88.82**
medBERT.de	75.35	75.35	75.35	85.71	83.33	84.51	80.13	84.03	82.03
BioGottBERT	72.07	72.07	72.07	83.33	<u>84.75</u>	84.03	80.77	84.68	82.68
GeistBERT	74.05	76.54	75.27	81.25	**88.14**	84.55	75.19	80.65	77.82
GeBERTa	75.35	75.35	75.35	85.71	83.33	84.51	80.13	84.03	82.03

Table S6: Overview of per entity precision (Prec.), recall (Rec.) and F_1 scores achieved on the BRONCO150 dataset All results are shown in percent and assess each model's best fine-tuned performance on the test set. The best model was selected out of 28 runs based on its validation set performance. Best score in bold and second best underlined.

Model	ActiveIng			Drug			Duration		
	Prec.	Rec.	F_1	Prec.	Rec.	F_1	Prec.	Rec.	F_1
ChristBERT	85.71	**92.96**	<u>89.19</u>	84.85	86.15	85.50	50.00	<u>60.00</u>	54.55
$\text{ChristBERT}_{\text{scratch}}$	85.62	90.14	87.82	84.38	83.08	83.72	**59.26**	58.18	<u>58.72</u>
$\text{ChristBERT}_{\text{BPE}}$	**88.52**	<u>92.40</u>	**90.41**	<u>91.14</u>	**91.14**	**91.14**	<u>58.82</u>	**60.61**	**59.70**
medBERT.de	85.93	90.06	87.95	88.89	88.89	<u>88.89</u>	46.97	49.21	48.06
BioGottBERT	86.29	90.85	88.51	87.30	84.62	85.94	50.85	54.55	52.63
GeistBERT	84.49	90.14	87.22	79.17	87.69	83.21	45.59	56.36	50.41
GeBERTa	<u>88.24</u>	89.55	88.89	**92.31**	<u>90.00</u>	**91.14**	55.17	50.79	52.89
	**Form**			**Frequency**			**Strength**		
ChristBERT	20.00	<u>25.00</u>	22.22	94.63	**96.04**	95.33	<u>97.10</u>	95.26	96.17
$\text{ChristBERT}_{\text{scratch}}$	**50.00**	**50.00**	**50.00**	93.10	93.56	93.33	**97.16**	<u>97.16</u>	**97.16**
$\text{ChristBERT}_{\text{BPE}}$	16.67	<u>25.00</u>	20.00	95.06	94.29	94.67	94.94	96.06	95.50
medBERT.de	**50.00**	**50.00**	**50.00**	95.00	<u>95.87</u>	95.43	95.15	96.86	96.00
BioGottBERT	**50.00**	**50.00**	**50.00**	<u>96.04</u>	**96.04**	**96.04**	95.37	**97.63**	<u>96.49</u>
GeistBERT	<u>33.33</u>	**50.00**	<u>40.00</u>	93.60	94.06	93.83	96.15	94.79	95.47
GeBERTa	**50.00**	**50.00**	**50.00**	**96.37**	95.60	<u>95.98</u>	96.03	96.41	96.22

Table S7: Overview of per entity precision (Prec.), recall (Rec.) and F_1 scores achieved on the CARDIO:DE dataset All results are shown in percent and assess each model's best fine-tuned performance on the test set. The best model was selected out of 28 runs based on its validation set performance. Best score in bold and second best underlined.

Model	Clinical			Diagnosis / Pathology			Diagnostic		
	Prec.	Rec.	F_1	Prec.	Rec.	F_1	Prec.	Rec.	F_1
ChristBERT	79.12	**84.28**	81.62	80.26	80.81	80.53	73.34	76.18	74.73
$\text{ChristBERT}_{\text{scratch}}$	79.87	83.60	**81.69**	80.35	**81.67**	**81.01**	**73.78**	76.82	75.27
$\text{ChristBERT}_{\text{BPE}}$	**80.14**	82.86	81.48	**80.66**	81.31	80.98	73.62	**77.54**	**75.53**
medBERT.de	76.29	81.02	78.58	78.46	78.88	78.67	72.09	74.19	73.13
BioGottBERT	79.14	80.99	80.05	78.47	79.82	79.14	73.15	74.46	73.80
GeistBERT	79.57	81.26	80.41	78.55	79.25	78.90	72.21	75.26	73.70
GeBERTa	79.81	83.08	81.42	80.39	81.22	80.80	72.93	77.09	74.95

Model	External Substance			Nutrient / Body Substance			Other Finding		
ChristBERT	56.47	53.93	55.17	**76.11**	72.11	**74.05**	67.35	67.01	67.18
$\text{ChristBERT}_{\text{scratch}}$	50.54	52.81	51.65	73.90	70.79	72.31	**68.78**	**67.88**	**68.33**
$\text{ChristBERT}_{\text{BPE}}$	57.43	**58.00**	**57.71**	70.74	71.43	71.08	68.45	67.71	68.08
medBERT.de	52.87	52.27	52.57	65.48	69.25	67.31	64.50	64.56	64.53
BioGottBERT	58.90	48.31	53.09	71.35	69.47	70.40	67.15	64.68	65.89
GeistBERT	55.42	51.69	53.49	69.17	**72.63**	70.86	65.17	64.36	64.77
GeBERTa	**59.49**	51.09	54.97	73.42	70.28	71.81	66.85	67.12	66.98

Model	Therapeutic		
ChristBERT	**79.55**	79.93	**79.74**
$\text{ChristBERT}_{\text{scratch}}$	79.06	**80.18**	79.62
$\text{ChristBERT}_{\text{BPE}}$	79.41	80.00	79.70
medBERT.de	77.09	77.41	77.25
BioGottBERT	78.01	78.63	78.32
GeistBERT	77.71	78.73	78.22
GeBERTa	78.85	79.03	78.94

Table S8: Overview of per entity precision (Prec.), recall (Rec.) and F_1 scores achieved on the GGPONC dataset All results are shown in percent and assess each model's best fine-tuned performance on the test set. The best model was selected out of 28 runs based on its validation set performance. Best score in bold and second best underlined.

Model	Trauma Surgery			Ophthalmology			Orthopedics		
	Prec.	Rec.	F_1	Prec.	Rec.	F_1	Prec.	Rec.	F_1
ChristBERT	84.21	**100**	**91.43**	100	100	100	89.19	**100**	94.29
$\text{ChristBERT}_{\text{scratch}}$	83.78	96.88	89.86	100	100	100	**96.97**	96.97	**96.97**
$\text{ChristBERT}_{\text{BPE}}$	82.35	87.50	84.85	100	100	100	91.67	**100**	95.65
medBERT.de	83.87	81.25	82.54	100	100	100	93.94	93.94	93.94
BioGottBERT	84.21	**100**	**91.43**	100	100	100	88.89	96.97	92.75
GeistBERT	**93.33**	87.50	90.32	100	100	100	88.57	93.94	91.18
GeBERTa	84.38	84.38	84.38	100	100	100	**96.97**	96.97	**96.97**
	**Emergency Medicine**			**Traumatology**			**Anesthesiology**		
ChristBERT	100	100	100	100	100	100	100	100	100
$\text{ChristBERT}_{\text{scratch}}$	100	100	100	100	100	100	100	100	100
$\text{ChristBERT}_{\text{BPE}}$	100	100	100	100	100	100	100	100	100
medBERT.de	100	100	100	100	100	100	100	100	100
BioGottBERT	100	100	100	100	100	100	100	100	100
GeistBERT	100	100	100	100	100	100	100	100	100
GeBERTa	100	100	100	100	100	100	100	100	100

Table S9: Overview of per class precision (Prec.), recall (Rec.) and F_1 scores achieved on the JSynCC dataset All results are shown in percent and assess each model's best fine-tuned performance on the test set. The best model was selected out of 28 runs based on its validation set performance. Best score in bold and second best underlined.
